# Is Oxidative Stress a Shared Mechanism Linking Autism Regression, Late Diagnosis, and Sex-Disparate Diagnostic Timing?

**DOI:** 10.3390/cells15212027

**Published:** 2026-11-06

**Authors:** George Ayoub

**Affiliations:** Psychology Department, Santa Barbara City College, Santa Barbara, CA 93109, USA; neuro@sbcc.edu

**Keywords:** autism spectrum disorder, developmental regression, oxidative stress, gut microbiome, folate metabolism, maternal immune activation, adverse childhood experiences, sex differences

## Abstract

**Highlights:**

**What are the main findings?**

- Early regression, late regression, gradual increases in symptom severity, and late diagnosis in autism differ in evidence base and may have different, partly overlapping explanations; diagnostic and recognition processes account for much of the late-diagnosis and sex-timing data.
- Environmental, infectious/autoimmune, and psychosocial exposures are associated with ASD risk and converge on oxidative stress and neuroinflammation in experimental work, but none has been linked to regression or late diagnosis; we present a hypothesis-generating model of cumulative burden, buffering capacity, and developmental timing, with explicit, testable predictions.

**What are the implications of the main findings?**

- Reducing avoidable environmental exposures and supporting families are reasonable general goals on independent grounds; this model does not imply that parents or families cause autism, and it does not support specific clinical interventions.
- Testing the model requires prospective, sex-stratified cohorts that measure oxidative stress, microbiome composition, folate/B12 status, cortisol, and exposure history together, a study design that does not yet exist.

**Abstract:**

Autism spectrum disorder (ASD) research has largely treated early regression, late decline after a period of stability, gradual increases in symptom severity, late diagnosis, and the sex difference in diagnostic timing as separate topics. These phenomena differ in definition, evidence, and plausible mechanism, and diagnostic and recognition processes may explain much of the late-diagnosis and sex-timing findings. This narrative, hypothesis-generating review asks whether a cumulative oxidative and inflammatory burden, arising from environmental chemical exposure, infection and autoimmune activity, and chronic psychosocial stress, and modified by genetic susceptibility, one-carbon metabolism, the gut microbiome, and individual buffering capacity, could contribute to some of these trajectories. We grade the evidence for each link in four tiers (human ASD data, other human data, experimental models, and our hypotheses). Most links rest on associations with ASD risk or on experimental data; no study has linked these exposures to regression or late diagnosis, and oxidative stress may be a consequence or correlate rather than a cause. We therefore define operational terms, derive predictions that could refute the model, and outline the prospective, sex-stratified evidence needed to test it.

## 1. Introduction

Autism spectrum disorder (ASD) is defined in DSM-5 by persistent deficits in social communication and interaction together with restricted, repetitive patterns of behavior, interests, or activities that begin in the early developmental period but may not become fully apparent until social demands exceed a person’s capacities [1]. DSM-5 replaced the earlier subtypes with a single ASD category defined by two symptom domains and treats the pattern of onset, including regression, as a descriptive specifier rather than a diagnostic criterion [1]; in a validation study the proposed criteria had higher specificity but lower sensitivity than the earlier criteria [2]. Research has historically treated several developmental phenomena within ASD as separate literature: early regression (loss of previously acquired skills, typically in toddlerhood) [3], the less common but increasingly documented “late regression” or symptom drift occurring after a period of relative developmental stability [4], and the sizable population of individuals, disproportionately female, who are not diagnosed until adolescence or adulthood, often without any documented childhood symptom loss [5]. These phenomena are typically studied with different methodologies, in different age brackets, and by largely non-overlapping groups of researchers.

We ask here whether some of these phenomena may share a contributing vulnerability: cumulative oxidative stress arising from the interaction of environmental exposures and psychosocial stress, possibly mediated in part by disruption of the gut microbiome, and consequent impairment of one-carbon metabolism. This is offered as a theoretical synthesis and hypothesis-generating framework rather than as an established causal account. No single study currently measures oxidative stress markers, microbiome composition, B-vitamin status, cortisol, and exposure history simultaneously in a cohort spanning early regression, late regression, and late diagnosis. What does exist is a set of adjacent, partially overlapping studies on regression epidemiology, PANS/PANDAS and autoimmune-triggered symptom change, adverse-childhood-experience (ACE) and trauma research in autism, sex-disparity in diagnostic timing, and an emerging literature on environmental chemical and dietary exposures as contributors to ASD risk. This paper’s contribution is to lay these studies alongside one another, propose a candidate mechanistic thread that could connect some of them, and specify what evidence would be needed to test the resulting model [6,7,8].

Because the environmental exposure literature has been reviewed in full in our recent pollutant-burden review [9], Section 3.2 of this paper summarizes those exposure classes only briefly and concentrates instead on how they bear on regression, late diagnosis, and the cumulative-threshold model.

Section 1.1, Section 1.2 and Section 1.3 state the aim, the literature search, and the evidence grading used. Section 2 defines the phenomena and the scope of the proposed mechanism. Section 3 addresses genetic susceptibility, psychosocial stress, environmental and infectious/autoimmune contributors to oxidative stress, and the endocannabinoid system as a candidate modulator. Section 4 evaluates maternal immune activation and related prenatal immune mechanisms, Section 5 the gut microbiome as a possible mediating pathway, and Section 6 psychosocial stress and cortisol. Section 7 reframes the sex disparity in diagnostic timing through this lens, Section 8 sets out an integrated hypothesis with operational definitions, predictions, and an evidence summary, and Section 9 addresses limitations and the evidence needed to test it.

### 1.1. Aim, Scope, and Relation to Prior Work

The aim of this review is to evaluate whether cumulative oxidative and inflammatory burden, modified by genetic susceptibility and by individual buffering capacity, is a plausible and testable contributor to certain ASD trajectories (early regression, late regression, and gradual increases in symptom severity) and, more tentatively, to differences in the timing of diagnosis, and to specify the evidence required to test it. We address four questions: (1) how are these phenomena defined, and how strong is the evidence for each; (2) which exposure and biological domains are associated with ASD, and at what evidence tier; (3) is there evidence that any of them converge on oxidative or inflammatory pathways in ways that bear on timing; and (4) which predictions would distinguish the model from alternative accounts, including diagnostic and masking explanations.

This review builds on our earlier work on critical periods, stress, and nutrition [6], genetic factors and inflammation [7], maternal immune activation, toxicants, and folate metabolism [8], and pollutant burden [9]. Those papers addressed ASD risk in general. The present review differs in focusing on timing and trajectory (regression and diagnostic timing), in adding infectious, psychosocial, and endocannabinoid considerations, and in defining a testable model with explicit predictions. Where it draws on the earlier reviews, the material is condensed and cited so that primary sources can be located, and we include evidence that argues against or limits the model (summarized in Section 9).

### 1.2. Review Design and Literature Search

This is a narrative, hypothesis-generating review, not a systematic review; no protocol was registered and no PRISMA-style screening was performed. Literature was identified mainly through PubMed, supplemented by Google Scholar and by the reference lists and search records of our earlier reviews [6,7,8,9], whose search approach is described in Section 1.1 of the pollutant-burden review. Search terms combined “autism” or “autism spectrum disorder” with terms for regression, developmental delay, diagnosis and diagnostic age, and sex differences, and with mechanism- and exposure-specific terms; further terms were added as the search progressed. A representative PubMed query, adapted for each topic, was as follows: (“autism spectrum disorder”[MeSH Terms] OR autis*[Title/Abstract]) AND (regression[Title/Abstract] OR “age at diagnosis”[Title/Abstract] OR “late diagnosis”[Title/Abstract] OR “sex differences”[Title/Abstract]) AND (“oxidative stress”[MeSH Terms] OR glutathione[Title/Abstract] OR “air pollution”[MeSH Terms] OR microplastic*[Title/Abstract] OR “maternal immune activation”[Title/Abstract] OR “adverse childhood experiences”[MeSH Terms] OR folate[Title/Abstract] OR microbiome[Title/Abstract]). Topic-specific searches replaced the third block with the relevant exposure or mechanism terms. Searches were not restricted by publication date, but we prioritized recent large cohorts, registry studies, and systematic reviews or meta-analyses. The most recent searches were conducted in September 2026. Records were screened by the author by title and abstract, and full texts were read for those bearing directly on a phenomenon in Table 1 or on a link in the proposed model. Studies were retained if they supplied evidence at one of the four tiers defined in Section 1.3 for such a link; within each topic we gave preference to systematic reviews, meta-analyses, and large prospective or registry studies, and cited smaller or single studies when they were the only evidence available, identifying them as such in the text and in Table 2. The reference list is therefore a purposive, not exhaustive, selection. Inclusion reflected the author’s judgment of relevance and quality; no formal risk-of-bias instrument was applied, and screening was not duplicated by a second reviewer. This approach risks selection bias and favors studies that fit the framework, which we try to offset by reporting null, conflicting, and confounded findings (Section 9).

### 1.3. Evidence Tiers and Terminology

Because the literature reviewed differ in design and relevance, we label the evidence for each link in the proposed model using four tiers. Tier 1 is human data in autistic people or children later diagnosed as ASD (cohort, registry, case–control, and trial data). Tier 2 is human data in other populations, or on exposure and biomarkers without an ASD outcome (for example placental biomonitoring, adult biomarker studies, and non-ASD clinical cohorts). Tier 3 is animal, organoid, or in vitro experiments. Tier 4 includes hypotheses and inferences proposed by the author for which no direct data exist. Support at one tier is not treated as support at another, and association with ASD risk is not equated with association with regression or late diagnosis. Tiers describe the type of evidence, not its quality, which is addressed in the text through design limits, null or conflicting results, and confounding. Terms follow Table 1: “regression” denotes loss of previously acquired skills; “late regression” denotes functional decline after a period of stability; “gradual increase in severity” (the “gained diagnosis” pattern) denotes rising symptom severity across development; and “late diagnosis” denotes the timing of recognition, not of onset. Three limits recur in this review and are stated here once rather than repeated in every section: associations with ASD risk do not establish causes of regression or late diagnosis; oxidative-stress markers may be a consequence or correlate of ASD rather than a cause (Section 3); and testing the model requires prospective, pre-onset sampling (Section 8.5 and Section 9). Later sections restate these limits only where they change how a specific finding should be read.

## 2. Phenomenology: Defining the Forms of “Regression”

Popular and clinical discourse often uses “regression” to describe several distinct phenomena. This section separates them, since the strength of evidence and plausible mechanism differs substantially across each. We also include later diagnoses, whether with some childhood symptoms (Section 2.3) or without (Section 2.4), so that the evidence bases and possible shared and distinct factors can be compared, without assuming that the phenomena share a mechanism.

### 2.1. Early Regression

The best-characterized form of regression is loss of previously acquired skills, most often expressive language, but sometimes social engagement, eye contact, or motor skills, occurring in a child who had developed typically for roughly the first year of life [3]. Most documented losses occur between 12 and 36 months, with onset clustering around 21–24 months, though at least one large epidemiological study found the window extends from as early as 6 months to as late as 7 years [7,10,11].

Prevalence estimates vary substantially by methodology. Retrospective parent-report studies commonly find regression in roughly one-third of ASD children, and a meta-analysis pooled this at approximately 32% [12,13]. Studies relying on medical or school records find a lower rate, near 21%, likely an undercount that depends on both a parent noticing the change and a clinician documenting it. Prospective studies, which track children over time rather than relying on retrospective report, find substantially higher rates: one study documented social-skill decline at some point between 6 months and 3 years in 86% of children later diagnosed as ASD, suggesting regression may be a far more common and gradual phenomenon than the classic “sudden loss” narrative implies [7,14]. Definitions also vary: regression is operationalized differently across studies, and caregivers can mistake a plateau in skills for regression, which may contribute to mixed findings on regression and later outcomes [1].

The mechanistic literature for early regression remains unsettled. Ozonoff and colleagues have proposed that apparent “loss” may not reflect a discrete neurological event at all, but rather a fading of skills from insufficient reinforcement of an underlying drive to seek out social communication [14]. Seizure or epileptiform activity has been investigated as a contributor but has not been consistently associated with regression in larger studies [15]. The MMR vaccine hypothesis, once prominent because the vaccine’s typical administration window (12–15 months) overlapped with common regression onset, has been thoroughly discredited and the originating study retracted; it is named here only to be set aside.

The clinical significance of distinguishing regression from non-regressed onset is reinforced by long-term outcome data: a population-based birth cohort study following 168 autistic individuals from early childhood into adulthood found that those with a history of regression had significantly poorer outcomes across multiple domains than those without, including lower adulthood IQ, higher incidence of epilepsy, and reduced composite social ability, even after accounting for early childhood IQ [16]. This is among the first studies to track regression status forward across decades rather than only characterizing it retrospectively, and it suggests that regression may mark a distinct, and prognostically meaningful, developmental subtype rather than merely a different route to a similar endpoint.

### 2.2. Late Regression After a Period of Stability

A smaller and much less studied literature addresses regression occurring after a period of relative developmental stability, a pattern distinct from, and in some ways the inverse of, early regression, since it implies a child who was previously doing comparatively well. Ghaziuddin’s case series remains the principal systematic account of this pattern: it describes marked functional decline emerging after stable childhood functioning, with a mean age of onset around 13 years. Obsessive slowing and ritualistic behavior were often the earliest signs, followed by mood and behavioral change, and catatonia was identified as a common feature, present in 17 of 20 cases, with outcomes in this cohort frequently poor. This literature is small, consisting of case series rather than a population-level epidemiological base, but it establishes adolescent-onset functional decline in autism as a real, if underexamined, clinical entity [4]. A more recent multicenter clinical sample extends this picture: among 300 autistic adolescents and young adults, catatonic presentation was closely tied to stressful life events, a finding that is compatible with, but does not by itself establish, a role for psychosocial stress as a precipitant of late regression, since stressful events can also follow from, or be reported differently in, a deteriorating clinical state. Catatonia and adolescent-onset decline may also reflect co-occurring psychiatric or medical conditions rather than a change in autism itself, a further reason not to assume that late regression shares a mechanism with early regression [17].

### 2.3. The “Gained Diagnosis” Trajectory

A related phenomenon, studied with more rigorous longitudinal methodology, is the trajectory Elias and Lord (2022) term “Gained ASD Diagnosis.” Following 155 individuals from age 2 to age 25 with blinded assessments at each wave, the authors identified four developmental trajectories, Retained ASD, Lost ASD, Never Had ASD, and Gained ASD Diagnosis, the last describing individuals whose adult diagnosis differed from, and typically exceeded, their childhood classification. Regardless of cognitive level, most of the newly met diagnoses appeared in adulthood, and among participants with IQ in the typical range the course was a slow rise in symptom severity scores rather than an abrupt relapse, so that diagnostic criteria were sometimes not clearly met until adulthood [18].

The authors interpret this as evidence that autism classification can genuinely shift across development, rooted in slow changes in core autism features that begin in childhood, a documented trajectory rather than simple diagnostic error, at least within this cohort. This remains a single strong longitudinal study rather than a settled consensus, and no mechanistic account yet explains why symptom severity drifts upward in some individuals during adolescence or adulthood, a gap this paper’s proposed model is intended to address [18]. Population-based data corroborate that this drift is not confined to clinical samples: a prospective UK birth cohort followed to age 25 identified a distinct “late-emerging” autistic-trait trajectory in a meaningful minority of the population, and a subsequent review of symptom-severity change across the lifespan likewise concludes that increasing, not only decreasing, severity trajectories are a recognized, if less common, pattern [19,20].

### 2.4. Late Diagnosis Without Documented Childhood Symptoms

A conceptually distinct but practically overlapping phenomenon is late diagnosis in individuals with no documented history of childhood symptoms at all, which may represent true late emergence, or simply late recognition of traits that were present but unrecognized. Definitional inconsistency is itself notable: a 2024 systematic review of 420 studies found only about a third specified a cutoff for “late” diagnosis at all, and those cutoffs averaged 11.5 years but ranged from age 2 to 55, with a bimodal distribution clustering around age 3 and age 18 [21].

Large cohort data underscore how common late diagnosis is: the SPARK cohort found roughly half of autistic adults were diagnosed after age 17, many not until their 30s, 40s, or 50s [5]. A case series documented six adult ASD diagnoses, showing the delay in diagnosis was related to healthcare access, co-occurring symptoms, and cultural/ethnographic data of the individual [22]. A Danish registry study is particularly relevant here: among individuals diagnosed as autistic when adults, 69% of men and 61% of women had no record of any related psychiatric or neurological diagnosis before age 18, a pattern the authors argue is difficult to explain as simple misdiagnosis or diagnostic overshadowing, and one that runs counter to the common assumption that autistic symptoms merely fade with age. Consistent with this, the Millennium Cohort Study found late diagnosis was predicted by typical-range intelligence and the absence of early parental concern, a profile consistent with a child who appears to be managing well until social and academic demands increase in adolescence [23,24]. Consistent with this pattern, a UK Millennium Cohort Study report focused specifically on mental health outcomes found that late-diagnosed children accumulate greater social and emotional difficulty across childhood and adolescence than their earlier-diagnosed peers, a trajectory of increasing burden that is compatible with, though not specific to, a cumulative-threshold framing, since greater difficulty after late diagnosis may equally reflect unmet support needs [25].

Late diagnosis also has well-documented explanations that do not require any change in underlying biology. Diagnostic criteria and screening tools have changed over time; people with higher cognitive ability, fewer behavioral concerns, or compensatory strategies are recognized later; access to assessment varies; co-occurring conditions such as anxiety, depression, and ADHD can lead to other diagnoses first; and the demands that expose difficulties often rise in adolescence and adulthood, so that a stable underlying profile may become visible only when the environment changes [21,22]. Because most studies of late diagnosis rely on registry or retrospective data, they cannot separate these processes from true emergence or worsening, and we therefore treat late diagnosis as the phenomenon least able to support mechanistic inference (Section 2.5).

For the purposes of this review, late regression (Section 2.2 and Section 2.3) and late diagnosis without documented childhood symptoms (Section 2.4) are treated as related but distinguishable phenomena. In both cases an individual not previously identified as meeting diagnostic criteria comes to meet them later, but the route may be genuine symptom emergence, gradual accumulation, delayed recognition, or a combination that the field currently has no reliable way to disentangle. We therefore do not assume a shared mechanism; the possibility that some of these phenomena share a contributing biological process is a hypothesis to be tested against the alternatives above.

**Figure 1:**
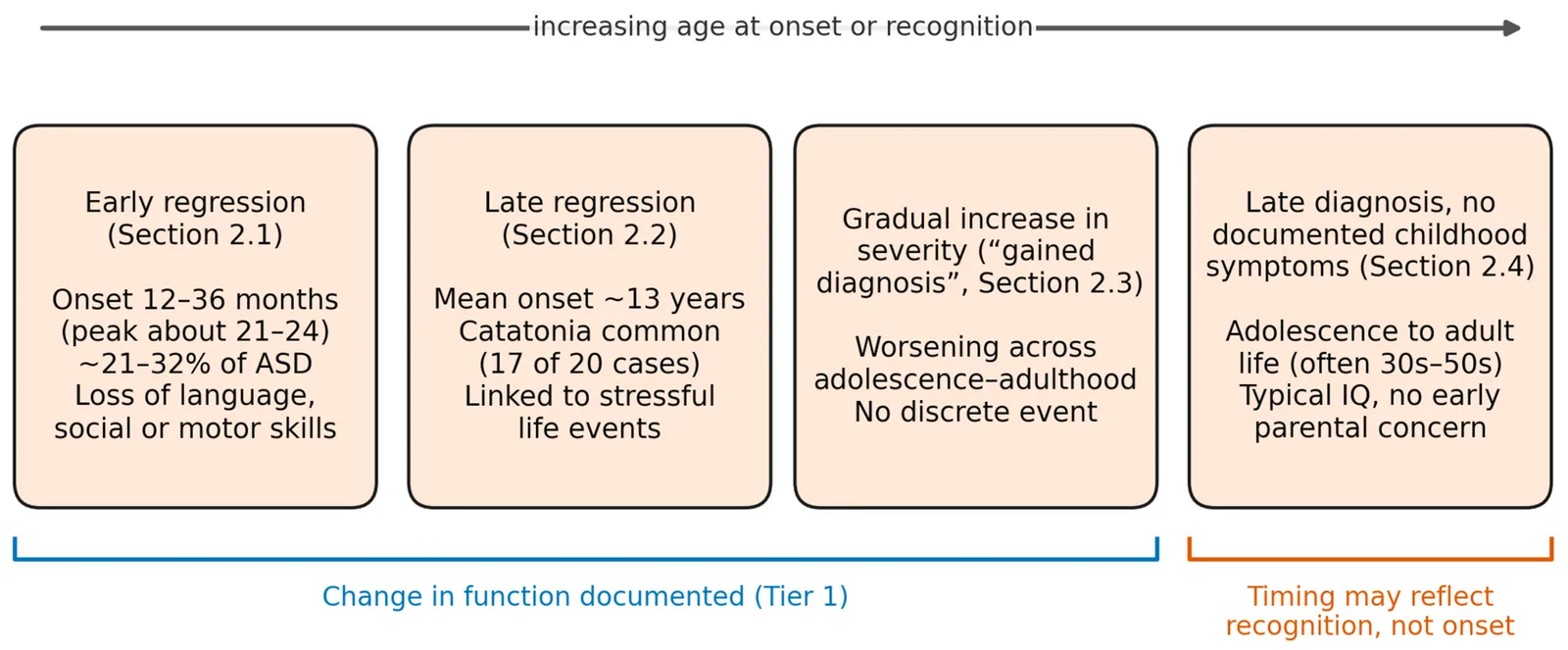
The four phenomenological patterns addressed (early regression, late regression, gained ASD diagnosis, and late diagnosis without documented childhood symptoms), ordered by the typical developmental timing at which the change or its recognition becomes clinically apparent. Early regression, late regression, and gradual increase in severity involve a documented change in function; the timing of late diagnosis may reflect recognition rather than onset. Line color indicates the evidence tier: blue, documented; brown, hypothesized.

Figure 1 summarizes these four patterns and the developmental timing that distinguishes them.

### 2.5. Scope of the Proposed Mechanism and Terminology

The phenomena in Section 2.1, Section 2.2, Section 2.3 and Section 2.4 differ in definition, in evidence base, and in how much their timing reflects biology rather than recognition (Table 1). We therefore do not propose a single process for all of them. The hypothesis developed in later sections is directed first at early regression, late regression, and gradual increases in severity, where a change in function is documented; late diagnosis without documented childhood symptoms and the sex difference in diagnostic timing are considered more tentatively, because recognition, masking, access, and changing criteria can account for much or all of the observed patterns. Different phenomena could also have different mechanisms (for example, an infection-triggered abrupt change versus a slow accumulation), so even where a shared pathway is hypothesized, we expect it to be one contributor among several.

**Table 1:** Clinical phenomena addressed in this review, with working definitions, evidence base, non-biological explanations, and relation to the proposed model.

Phenomenon	Working Definition	Main Evidence Base	Non-Biological or Alternative Explanations	Relation to the Proposed Model
Early regression	Loss of previously acquired skills (usually language or social engagement), typically at 12–36 months, after apparently typical early development	Retrospective parent report; records-based studies; prospective cohort data; one population birth-cohort follow-up	Recall bias; gradual fading rather than abrupt loss; detection depends on a parent noticing and a clinician documenting	Primary target for hypothesis testing (Section 8.2)
Late regression (adolescent-onset decline)	Marked functional decline after a period of stable functioning, mean onset about 13 years, often with catatonic features	Case series; one multicenter clinical sample	Catatonia and co-occurring psychiatric or medical illness; small clinic-based samples	Primary target, with lower confidence
Gradual increase in symptom severity (“gained diagnosis”)	Rising ASD symptom severity across development, so that criteria are met later	One longitudinal cohort; population-cohort trajectory analyses	Measurement change across development; shifting demands; instrument sensitivity	Possible target
Late diagnosis without documented childhood symptoms	Diagnosis in adolescence or adulthood with no recorded childhood symptom loss	Registry, population-cohort, and large clinical datasets	Delayed recognition; masking; access to assessment; comorbidity; changed criteria	Not a primary target: biology cannot be inferred from timing alone
Sex-disparate diagnostic timing	Later median age at diagnosis and a larger adult-diagnosed share in females	Records data; national registries	Differences in presentation and criteria; masking; referral patterns	Hypothesis only (Section 7.3), complementary to the masking account

### 2.6. Genetic Etiology and Molecular Architecture of ASD

ASD is among the most heritable neurodevelopmental conditions, with heritability estimates of roughly 65–90% and a recurrence risk of about 8-fold for siblings [26,27], so any environmental or physiological model must be read alongside a large genetic contribution (Tier 1). Cases are commonly classed as syndromic (autism as part of a defined genetic or chromosomal syndrome, often with intellectual disability, dysmorphic features, or epilepsy), non-syndromic (no recognizable syndrome, although pathogenic variants may still be found), or idiopathic (no single genetic or environmental cause identified) [26], although the boundary between rare monogenic and non-syndromic forms is not sharp [27]. The architecture combines chromosomal abnormalities, copy number variants (CNVs), and single-nucleotide variants, arising de novo or inherited, with a polygenic background of many common variants of small effect; neither rare nor common variants alone account for risk, and environmental and epigenetic modifiers are thought to interact with this background [26]. Some CNV effects follow a two-hit pattern, in which two alterations together produce a phenotype that neither produces alone [27].

Molecular diagnosis remains limited: reported yields are about 9–20% for chromosomal microarray, 10–30% for exome sequencing, and 20–40% for genome sequencing, so most cases have no identified molecular cause [26]. Risk genes (for example *CHD8*, *ADNP*, *ARID1B*, *SHANK3*, *SYNGAP1*, *SCN2A*, *GRIN2B*, *FOXP1*, and *DYRK1A*) converge on transcriptional regulation, chromatin remodeling, synaptic function, and neuronal signaling [26]. The average male-to-female ratio is 4–5:1, and the female protective effect model proposes that sex steroid hormones and sex-differentiated neural cell activity contribute [27]; in one study summarized in the review, ASD females more often carried highly penetrant CNVs [26].

This genetic architecture bears on the present review in three ways. First, it limits what any exposure-based model can claim: many cases of regression or late diagnosis will have a substantial genetic basis, and syndromic forms with regression exist, so the hypothesis developed here is at most one contributor among several. Second, genetic susceptibility is the substrate for the modifiers discussed in Section 3.1 and Section 5.3 (folate-pathway, detoxification, and mitochondrial genes) and for the buffering-capacity construct of Section 8.5; the two-hit logic of CNVs resembles the multi-hit exposure logic of Section 3.4, but it is separate evidence (Tier 1) from the proposed exposure-based thresholds (Tier 4). Third, sex-linked genetic effects are relevant to Section 7, where they are an alternative to, or companion of, the proposed sex differences in oxidative buffering.

## 3. Oxidative Stress and Environmental Contributors

Oxidative stress, an imbalance between reactive oxygen species production and antioxidant capacity, has been repeatedly documented in ASD populations, including reduced glutathione levels and altered markers of oxidative damage relative to neurotypical peers. Whether oxidative stress is a primary driver, a downstream consequence of other biological or genetic alterations, or a correlate of diet, medication, sleep, or illness in these samples remains unresolved; we therefore treat it as a candidate point of convergence, not as an established cause (Section 8.5).

### 3.1. Rationale: Oxidative Stress as a Developmental Vulnerability

The developing brain is thought to be particularly vulnerable to oxidative insult given its high metabolic demand, relatively high lipid content, and comparatively immature antioxidant defense systems in early life. This vulnerability is not confined to infancy: puberty and adolescence involve substantial neurodevelopmental remodeling and correspondingly elevated metabolic demand, offering a plausible second window of heightened sensitivity, one that aligns temporally with the late-regression pattern described in Section 2.2 (mean onset around age 13) [6,7].

For the purposes of this review, what matters about the exposure classes in Section 3.2 is less their individual mechanisms than where those mechanisms might meet. Each acts through some combination of oxidative and mitochondrial injury, barrier breakdown, neuroinflammation with disturbed microglial pruning, endocrine interference, and epigenetic change, and we treat them as candidate routes into a shared pathway, a convergence argued in our pollutant-burden review (Section 3 of [9]). The step of the developmental sequence affected (neurogenesis, corticogenesis, synaptogenesis, or activity-dependent pruning) would depend on the identity, dose, and timing of the exposure, within windows of heightened sensitivity [6]. The similar social-communication and repetitive-behavior phenotype is then proposed to reflect disruption of the same developmental program, an inference that is itself a hypothesis (Tier 4). This is what would justify pooling heterogeneous exposures into the single cumulative-burden model developed below.

Individual susceptibility to this oxidative burden is not uniform, and genetic variation in folate-pathway and mitochondrial genes offers one explanation for why comparable exposure produces regression, or ASD more broadly, in one child and not another.

Three genes illustrate the folate-pathway component, and they differ markedly in effect size and in the strength of evidence. Folate receptor alpha (FRα, encoded by *FOLR1*) is a high-affinity receptor that transports folate across the choroid plexus and placenta into the central nervous system. The reduced folate carrier (RFC, encoded by *SLC19A1*) is a more widely expressed, lower-affinity transporter that provides an alternative route for reduced folates. Methylenetetrahydrofolate reductase (MTHFR) converts 5,10-methylenetetrahydrofolate to 5-methyltetrahydrofolate, the form used to remethylate homocysteine to methionine. Biallelic *FOLR1* loss-of-function mutations are rare and cause a severe monogenic cerebral folate deficiency syndrome with early-onset neurological features, whereas heterozygous carriers and most other *FOLR1* variants have a much less certain and likely small effect. By contrast, *SLC19A1* rs1051266 and the common *MTHFR* variants (C677T and A1298C) are frequent polymorphisms with modest population-level effects on folate handling; case–control and meta-analytic findings in ASD are heterogeneous across populations, and none establishes a causal role [9]. Their relevance here is as small-effect susceptibility factors that may combine with pollutant, infectious, or psychosocial exposure, not as equivalents of a rare pathogenic *FOLR1* mutation.

A separate genetic vulnerability, involving mitochondrial function rather than folate transport, bears more directly on the regression phenotype addressed throughout this paper: the sequence of dysfunction differs by regression status, with inflammation and immune dysregulation appearing first in non-regressed children, but mitochondrial dysfunction appearing first in children who go on to regress, with any effect on immune activity emerging only later [7]. This is a single-study observation in a clinically heterogeneous group, and we are not aware of independent replication of the temporal ordering, so it should be treated as provisional (Tier 1, single study). It raises the possibility that mitochondrial genetic vulnerability constitutes a distinct, and possibly earlier-acting, susceptibility factor specific to the regression subtype, though the oxidative burden reviewed in Section 3.2 could plausibly stress mitochondrial function directly and interact with this vulnerability rather than acting independently of it. Cord blood metabolomics offers one emerging, non-invasive biomarker candidate for this pathway: machine-learning analysis of cord blood metabolic profiles has proven more predictive of later ASD diagnosis than maternal mid-gestational blood in both male and female offspring, and elevated cord blood acylcarnitine specifically predicts both ASD and ADHD outcomes, though neither finding has yet been tested against the environmental exposures reviewed here [7].

#### 3.1.1. Emotional and Psychosocial Stress

Emotional and psychosocial stress belongs alongside the chemical, dietary, and infectious/autoimmune contributors addressed elsewhere in this section as a distinct route to oxidative burden, rather than a phenomenon confined to Section 6’s discussion of family stress and cortisol. Maternal stress during pregnancy elevates cortisol and pro-inflammatory cytokines in ways that plausibly disrupt fetal brain development directly, and chronic stress in early childhood carries a parallel risk for immune and neurological outcomes. Emotional stress is therefore proposed to act on the same oxidative-stress axis as the chemical and infectious exposures discussed in this section, rather than as a separate psychosocial category standing apart from them, a family under chemical and psychosocial stress simultaneously is, on this model, accumulating oxidative burden along convergent rather than independent pathways [6,7]. Evidence status: the maternal-stress findings come largely from non-ASD or animal data (Tier 2–3); direct human evidence linking psychosocial stress to oxidative burden in ASD, or to regression, is lacking (Tier 4).

#### 3.1.2. Adverse Childhood Experiences and Prolonged HPA-Axis Activation

Adverse childhood experiences (ACEs), meaning abuse, neglect, household dysfunction, food or housing insecurity, or other chronic family stress that persists through childhood rather than occurring in a single prenatal window, plausibly constitute a distinct route to oxidative burden from the acute maternal-stress pathway described in Section 3.1.1. The foundational epidemiology here is a large pediatric dose–response relationship: a study of children and adolescents enrolled in an addiction, mental health, and psychiatry program found that specific ACE survey items were systematically associated with specific ICD-10 psychiatric diagnostic categories, with cumulative ACE burden tracking cumulative psychiatric risk [28], a pattern consistent with the general population dose–response relationship long established by the original ACE Study and its successors, though this particular cohort was not autism-specific.

Chronic childhood adversity is thought to become biologically embedded rather than remain a purely psychological exposure. In a 17-year prospective study, trauma-related DNA methylation risk scores derived from blood predicted adult depression, externalizing problems, and substance use more accurately than reported trauma history [29], and neuroimaging in the Adolescent Brain Cognitive Development cohort identified a neural signature shared by genetic and environmental risk for mental illness [30], a pattern parallel to the convergence argument of Section 3.4 but applied to psychosocial rather than chemical exposure.

The link to oxidative and immune pathways is more direct but indirect for ASD: in adults with major depression, adverse childhood experiences, atherogenicity, and an immune-linked neurotoxicity composite were significantly interrelated, with ACE burden explaining a meaningful share of variance in the composite [31]. This supports treating chronic ACE burden as an immune- and oxidative-stress-relevant exposure, but the evidence comes from an adult depression cohort, not from autistic or pediatric samples (Tier 2).

ACE-driven burden differs from the acute maternal-stress pathway in duration: acute prenatal stress is a bounded exposure window, whereas chronic ACE exposure over months or years could produce a cumulative, potentially self-reinforcing burden through epigenetic and neuroimmune mechanisms, more closely resembling the cumulative-threshold model of Section 3.4. The parallel clinical literature on ACEs in autistic populations, including prevalence data and the CASSQ instrument, is addressed in Section 6.2 and should be read together with this section.

ACE-related vulnerability may not require the child’s own exposure. In a 2025 clinical sample of 116 caregivers presenting children aged 0–5.9 years to a German mental health clinic, parental ACE burden was elevated relative to the general population, and parental neglect was associated with child adjustment disorder diagnosis (OR = 2.54) [32]. The authors discuss psychosocial transmission (attachment, caregiving, stress reactivity) alongside biological routes, including lasting hypermethylation of the glucocorticoid-receptor gene *NR3C1* reported after childhood maltreatment. Whether such intergenerational mechanisms bear on neurodevelopment or autism is untested; none of the studies cited in this section examined ASD outcomes (Tiers 2–4), and we raise the idea only as a hypothesis that parental history could layer onto a child’s own exposures.

### 3.2. Chemical and Dietary Exposure Categories

The exposure classes in this section are reviewed in full in our recent pollutant-burden review [9]. Because evidence quality differs widely between classes, each subsection states the strongest human evidence, conflicting or null findings, principal confounders and uncertainties, and what the evidence does and does not say about regression or late diagnosis. Two general limits apply throughout. First, with few exceptions, outcomes are ASD diagnosis or autistic traits, not regression (Section 1.3). Second, most studies measure exposure in pregnancy or infancy, whereas the phenomena in Table 1 occur later, so any link to later decline would require additional, untested assumptions about delayed or cumulative effects. Evidence strength also reflects which cohorts happened to measure which exposures as much as biological plausibility, and the classes should not be read as ranked.

#### 3.2.1. Micro- and Nanoplastics

Micro- and nanoplastics (MNPs) are now detectable in essentially every human placenta examined and in post-mortem human brain tissue, where concentrations substantially exceeded those in paired liver and kidney [33,34,35,36]. Human placental explant and cord-blood data additionally tie particle burden to oxidative stress and to altered fetal glucocorticoid and androgenic hormone levels [37,38]. In experimental systems, two partly separable developmental targets emerge: impaired corticogenesis, seen in human cortical spheroids [39] and in rat offspring exposed during gestation and lactation [40], and disrupted microglia-mediated synaptic pruning, seen after neonatal exposure in mice, with social-behavior deficits that persisted into adulthood [41]. General neurotoxicity reviews describe a recurring pattern of blood–brain barrier penetration, oxidative stress, mitochondrial dysfunction, and chronic neuroinflammation [42,43].

Studies closer to ASD include a mouse model in which prenatal polyethylene microplastic exposure produced social and repetitive-behavior changes alongside ASD-associated microbiome shifts [44] and a CHARGE cohort analysis in which parental occupational exposure to plastics and polymers was associated with greater symptom severity, rather than diagnosis risk, in ASD children [45]. A rat model also implicates P53-mediated ferritinophagy and ferroptosis in the offspring hippocampus after maternal nanoplastic exposure [46]. Ferritinophagy is the autophagic breakdown of ferritin to release iron [47] and ferroptosis is an iron-dependent form of cell death driven by lipid peroxidation [48]. Both processes are implicated in neurodegenerative and mitochondrial-vulnerability pathways more generally, which suggests that MNPs may act as an upstream trigger for previously described downstream mechanisms rather than as a wholly distinct one. Lifelong exposure beginning in utero also produced an ADHD-like phenotype in mice [49]. Evidence status: human data establish exposure (detection in placenta and brain; Tier 2), but analytical methods give concentrations that differ by orders of magnitude between laboratories, brain detection data are correlational, and no study has related MNP burden to an ASD outcome. The neurodevelopmental and ASD-like findings come from animal and in vitro models (Tier 3), often with polystyrene particles and doses whose relevance to human exposure is uncertain, and the CHARGE finding concerns severity in already-diagnosed children with exposure assigned from parental occupation. MNPs therefore illustrate a plausible mechanistic route, not an established risk factor, and nothing links them to regression (Tier 4); the full evidence is presented in Section 2.1 of [9].

#### 3.2.2. Endocrine-Disrupting Chemicals and Tire Wear Particles

Plastics are manufactured with, and leach, endocrine-disrupting chemicals (EDCs), chiefly phthalates, bisphenol A (BPA), and organophosphate esters (OPEs). Their human epidemiology is more mature than that of particles alone, although individual cohort findings are often modest or inconsistent. The best-worked example links higher prenatal BPA to clinician-confirmed ASD in boys with a genetic profile conferring low brain aromatase activity, with a mechanism traced through aromatase suppression and amygdala dysfunction in mice [50] and a multi-cohort ECHO analysis associated specific OPE metabolites with higher autistic-trait scores and diagnosis odds, more strongly in males [51]. The EDC literature points most consistently toward thyroid-hormone disruption in the second and third trimesters, and a 2024 mechanistic review proposes that phthalate exposure and micronutrient (folate and B-vitamin) status jointly disrupt prenatal one-carbon and lipid metabolism [52] a link to the folate-pathway vulnerability described in Section 3.1 that, to our knowledge, has not been tested directly in an ASD-relevant cohort. Evidence status: the BPA–aromatase finding is the best-supported mechanistic example, but it applies to a genetically defined subgroup of boys in one cohort. Elsewhere the findings are inconsistent: a mixture analysis of two cohorts reported different patterns in a general-population cohort and a cohort with higher familial likelihood of autism [53], a meta-analysis called the phthalate evidence slight and indeterminate [54], and an analysis in a cohort enriched for familial ASD likelihood found no clear effect of gestational phthalates on later behavior [55]. Exposure misclassification, correlated mixtures, sex-specific effects, and outcomes that capture general behavior rather than ASD limit interpretation; see Section 2.2 of [9] for the cohort-level evidence.

Tire wear particles are a dominant source of environmental microplastics and carry a distinct chemical hazard: the tire antioxidant 6PPD reacts with ozone to form 6PPD-quinone, which crosses the placenta in pregnant mice, reaches the fetal brain at concentrations several-fold above its parent compound, and activates nuclear-receptor pathways involved in neurodevelopmental gene regulation [56,57]. Because tire-derived and combustion-derived particles share vehicular traffic as a source, roadway-proximity tire-particle exposure may partly underlie the prenatal PM2.5–ASD association discussed below, a hypothesis that remains untested (see Section 2.4 of [9]). Evidence status: the 6PPD-quinone placental-transfer findings come from mice (Tier 3), human exposure data are limited, and no human ASD data exist, so the tire-particle hypothesis is a Tier 4 proposition.

#### 3.2.3. Ambient and Indoor Air Pollution

Air pollution has the largest and most consistent human evidence base of the exposure classes considered here. Meta-analytic and large-cohort data associate gestational PM2.5 with a roughly 15% higher ASD risk, largest in the third trimester, with exposure in the second postnatal year carrying the highest risk of any window examined. Cohorts that resolved PM2.5 into its components implicate different leading constituents in different populations, such as sulfate and ammonium in Ontario and NO2 and SO2 in South Korea, which points to source-specific mixtures converging on a shared oxidative and inflammatory pathway rather than a single causal pollutant [58,59,60]. A CHARGE analysis of size-resolved particles down to the ultrafine fraction found first-year exposure to iron, manganese, black carbon, and sodium associated with higher ASD odds [61]. The timing of these associations, spanning late gestation and early postnatal life, is consistent with the critical-window framing used in this paper. Evidence status: this class has the largest human ASD evidence (Tier 1), but effect sizes are modest, the leading components differ between cohorts, and unmeasured confounding by socioeconomic position, urbanicity, co-exposures, and diagnostic ascertainment is likely.

Wildfire smoke adds a distinct source. A California study of 8.6 million births associated wildfire-related PM2.5 with up to a 50% increase in ASD odds in otherwise low-pollution areas, and gestational smoke exposure shifted maternal circulating oxylipins, inflammation-related lipid mediators, for up to 13 months, offering a plausible peripheral-inflammatory mechanism [62,63]. Indoor sources (gas stoves, mold, and water damage) have also been studied in a large ECHO sample, where effects on birth outcomes were mixed and subgroup-specific [64]; household exposure reduction may therefore matter independently of ambient air-quality standards. Two routes could explain why such varied sources converge on the same outcome: oxidative injury to fetal neural tissue from redox-active metals carried on ultrafine particles, and maternal systemic inflammation whose cytokine and oxylipin signals reach the fetus without particles entering fetal tissue. We treat both as inputs to the cumulative burden in our model; the cohort data and mechanistic detail are in Section 2.3 of [9]. Evidence status: the wildfire finding is notable for a stronger association in otherwise low-pollution areas, which suggests that background exposure and selection can obscure associations, and the indoor-air data are mixed.

#### 3.2.4. Heavy Metals, Pesticides, and Industrial Chemicals

The metals, pesticide, and persistent-pollutant literature is the oldest and least uniform of the exposure classes considered here, and for this review it matters chiefly through its timing and susceptibility signals rather than through any single strong effect. A systematic review found associations between toxicant burden and ASD in most of the studies it examined, but the largest cohort-restricted meta-analysis graded overall certainty as low to very low once heterogeneity and publication bias were considered, and we adopt that caution [65,66]. The more specific findings concern household pesticides, where professionally applied indoor insecticides were linked to about twice the odds of ASD and flea and tick products to poorer cognitive and adaptive functioning [67,68], and fetal-exposure biomarkers, where persistent organic pollutants measured in tooth dentine pointed to the third trimester and to boys [69], echoing the late-gestation and male-vulnerability themes noted for the other exposure classes. In this class, genetic capacity for detoxification (for example *PON1* and the glutathione S-transferase genes), rather than dose and timing alone, appears to shape individual susceptibility; study-level detail is given in Sections 2.5 and 2.6 of [9]. Evidence status: associations are Tier 1 but come mainly from case–control designs and a few cohorts, which are more open to recall and selection bias, the dentine finding awaits independent replication, and exposure to metals, pesticides, and industrial chemicals is correlated with socioeconomic and residential factors that were not uniformly controlled.

#### 3.2.5. Ultraprocessed Food Intake

Ultraprocessed food (UPF) intake is a dietary pattern rather than a pollutant exposure. It is included because it may drive the same downstream oxidative stress and systemic inflammation and could therefore add to cumulative developmental risk. The strongest causal evidence is a small NIH inpatient trial in which participants consumed roughly 500 kcal/day more on an ultraprocessed diet than a matched unprocessed diet despite controlled presented calories and macronutrients, demonstrating that ultra-processing itself can drive excess intake independent of nutrient content [70]. Observational work links higher UPF intake to elevated C-reactive protein in adults and to higher urinary 8-hydroxy-2′-deoxyguanosine, a marker of oxidative DNA damage, in adolescents [71,72] a biomarker study in older adults with metabolic syndrome points the same way [73] and mechanistic reviews propose a cascade from emulsifier- and sweetener-driven gut-barrier disruption through endotoxin translocation to systemic inflammation and mitochondrial oxidative stress [74]. Two non-exclusive routes could connect this pattern to the folate framework developed here: maternal systemic inflammation arising from metabolic endotoxemia and crossing the placenta by the route proposed for the maternal-stress pathway, and displacement of nutrient-dense foods rich in folate, B vitamins, and other antioxidants, which could raise oxidative burden while lowering the nutritional buffering capacity needed to counter it. No study to date has tested either route directly in a pregnant or ASD-relevant cohort, so UPF should be read as a plausible, testable extrapolation rather than an established prenatal risk factor (see Section 2.7 of [9]). The randomized evidence concerns energy intake, not inflammation, and the biomarker studies are cross-sectional (Tier 2) and only partly independent of adiposity.

### 3.3. Infection- and Autoimmune-Associated Change

Three different kinds of change are discussed under this heading and should not be conflated: (a) abrupt neuropsychiatric symptoms such as obsessive–compulsive symptoms, food restriction, anxiety, and emotional lability, as defined for PANS; (b) loss of previously acquired developmental skills, the usual meaning of regression in autism (Section 2.1); and (c) worsening of existing autism symptoms or of function. PANS is defined by abrupt onset of obsessive–compulsive symptoms or severely restricted food intake accompanied by at least two additional cognitive, behavioral, or emotional symptoms that are not better explained by another disorder, and developmental or behavioral regression is one of several optional accompanying symptom categories; PANDAS is the subset temporally associated with group A streptococcal infection [75]. PANDAS was proposed in 1998 on five working criteria: OCD and/or a tic disorder, prepubertal onset, an episodic course with abrupt onset or dramatic exacerbations, association with group A streptococcal infection, and association with neurological abnormalities [76,77]. Because not all cases are tied to streptococcus, and viral infections or other illnesses can also precede exacerbations [78], the broader label PANS was proposed in 2012, with criteria that drop the infection requirement, tics as a primary criterion, and the prepubertal restriction and that emphasize abrupt OCD or restricted food intake; the stated aim was to define the cohort more narrowly and to standardize research [76,77]. It remains unclear whether the PANS criteria delineate a distinct clinical entity, PANDAS is not part of DSM-5, and definitive proof of an autoimmune mechanism is lacking [77,78]. Diagnosis rests on clinical judgment and a retrospective history of onset, and criteria and terminology have evolved, so the label does not by itself establish a distinct disease entity or a loss of acquired skills.

The literature on abrupt neuropsychiatric change attributed to infection or immune activation is the most directly relevant existing body of evidence for the “stable baseline, then sudden decline” pattern this paper is concerned with, and it deserves particular weight in this framework. Pediatric Acute-onset Neuropsychiatric Syndrome (PANS) and its streptococcus-specific subtype, Pediatric Autoimmune Neuropsychiatric Disorders Associated with Streptococcus (PANDAS), describe an abrupt change in neuropsychiatric symptoms that is thought to follow an infection-provoked, misdirected immune response and resulting brain inflammation in a genetically susceptible child [75]. The defining feature of PANS/PANDAS, a sudden shift from a relatively stable baseline, is precisely the pattern that distinguishes late regression (Section 2.2) from the more gradual symptom drift described for the “gained diagnosis” trajectory (Section 2.3). In autistic children specifically, the degrees of overlap with PANS/PANDAS remains poorly quantified: existing estimates range from clinical impressions of a small subset up to case series suggesting a substantial minority, but no population-based prevalence study has yet isolated PANS/PANDAS rates within an autistic cohort specifically, and existing figures should be treated as provisional pending better controlled research [79].

This literature suggests a plausible but untested oxidative-stress-adjacent mechanism within the regression literature: infection-triggered neuroinflammation is itself thought to involve substantial oxidative processes, and autoimmune/inflammatory activation is one of the more plausible convergence points between an infectious trigger and the broader chemical-exposure and psychosocial-stress pathways addressed elsewhere in this paper. If environmental chemical burden and family stress independently elevate baseline oxidative and inflammatory tone (Section 3.2 and Section 6), a subsequent infection may be more likely to precipitate a PANS/PANDAS-type regression in a child already operating with reduced antioxidant reserve, a multi-hit interaction that has not, to our knowledge, been directly tested. Some indirect support comes from a small biomarker study finding that autism spectrum status was associated with altered folate-receptor autoantibody binding within a PANS/PANDAS-diagnosed cohort, consistent with shared immune pathways, though the sample size (*n* = 47) and lack of a non-ASD PANS/PANDAS comparison group limit what can be concluded [80].

Recent large-cohort and clinical-consensus work has sharpened the epidemiology of this pattern and the case for infection as a trigger of neuropsychiatric change. A 2023 retrospective review of over 90,000 pediatric patients across three academic medical centers put the yearly incidence of PANDAS/PANS at roughly one case per 11,765 children aged 3 to 12 [79], a considerably more conservative figure than some of the wider community-derived estimates, and the American Academy of Pediatrics’ first clinical report specifically addressing PANS, published in 2025, now provides consensus diagnostic and management guidance, reflecting the condition’s move from a contested diagnosis toward mainstream clinical recognition [81]. Direct causal evidence that infection can precipitate neuropsychiatric events in children, as opposed to a coincidental association (though not specifically in PANS/PANDAS or autism), comes from a 2025 study showing that pediatric influenza infection itself, not antiviral treatment, drove an increase in neuropsychiatric hospitalizations, and that oseltamivir treatment suppressed this increase, isolating the infection as the causal trigger rather than treatment-related confounding [82]. A parallel mechanistic account, still preprint but directly relevant to this paper’s oxidative-and-inflammatory-burden framework, proposes that maternal immune activation imprints a lasting regulatory T-cell deficiency in offspring, removing a key brake on neuroinflammation and creating a durable susceptibility to autism-like phenotypes that could plausibly also lower the threshold at which a later infection precipitates PANS/PANDAS-type regression [83].

Temporal associations between infection and symptom change are open to alternative explanations: ascertainment (families and clinicians are more likely to notice and report change after an illness or hospital visit), reverse causation (prodromal symptoms increasing susceptibility to or detection of infection), shared genetic or immunological susceptibility, stress or medication effects, and the high background frequency of both infections and behavioral fluctuation in children. The strongest causal evidence, the influenza natural-experiment study, concerns neuropsychiatric hospitalization in the general pediatric population, not regression in autism. The PANS/PANDAS literature therefore supports the plausibility of an infection-triggered abrupt change in some children (Tier 1–2), but not yet a role in autism regression or an oxidative mechanism (Tier 4). In a Danish cohort of more than one million children, the incidence rate ratio for OCD, tics, or other mental disorders was 1.16 after a positive streptococcal test and 1.07 after a negative test, each compared with children never tested [77]; such modest associations, present even after negative tests, are compatible with ascertainment or non-streptococcal infections as well as with streptococcal causation. The only randomized trials of immunotherapy found little difference from placebo, and responses to standard psychiatric treatment resemble those in OCD without PANS criteria [77], so the autoimmune mechanism remains unproven.

The same MIA/autoimmune literature converges with the pollutant burden discussed in Section 3.2: maternal infection and inflammation, environmental toxicant co-exposure, maternal autoimmune disease, and folate receptor alpha autoantibody-mediated cerebral folate deficiency have been proposed as four overlapping routes into one downstream cascade of neuroinflammation, oxidative stress, mitochondrial dysfunction, and disrupted synaptic pruning (Section 6 and Figure 1 of [8]). Under that framework, PANS/PANDAS would be best understood as one clinically recognizable instance of a postnatal infection precipitating regression in a child whose neuroimmune system is already operating with reduced buffering capacity, whether from prenatal chemical burden, folate-pathway genetic vulnerability, or antecedent maternal immune or autoimmune exposure. The prenatal immune mechanisms themselves are summarized in Section 4.

### 3.4. Convergent, Multi-Hit Exposure Logic

Our central working hypothesis is that no single exposure category, chemical, dietary, infectious, or psychosocial, is likely to be sufficient on its own to explain regression or late diagnosis in most individual cases. Rather, the hypothesized model treats these exposures as additive or synergistic contributors to a cumulative oxidative burden (whether they are in fact additive, interactive, or threshold-like has not been tested; Section 8.5), such that regression or symptom emergence occurs when that burden crosses an individual threshold, one that is presumably shaped by genetic antioxidant capacity, baseline exposure history and, as discussed in Section 7, possibly sex. Under this framing, PANS/PANDAS-type infection-triggered regression is not a competing explanation to an environmental-chemical or psychosocial-stress account, but a possible precipitating final event layered onto a baseline burden shaped by the other pathways.

This convergence logic is not only our own synthesis; it follows the conclusion of our earlier review of the maternal immune activation, autoimmune, and environmental toxicant literature (Section 6 of [8]). A large systematic review and meta-analysis of cohort studies similarly finds that environmental pollutant exposures function as risk modifiers rather than independent sufficient causes, an ASD-risk finding compatible with a cumulative, multi-pathway account [66]. Extending that account to threshold crossing and regression is this paper’s hypothesis and has not been tested.

This convergence framing carries an important epistemic caveat that we acknowledge explicitly: a recent large population-based analysis examining maternal immune activation and autism causation directly addressed the question of whether the MIA-ASD association reflects a genuine causal pathway or is substantially attributable to familial confounding, genetic and environmental factors shared between a mother’s health status and her child’s neurodevelopmental outcome, rather than direct in utero exposure effects [84]. This does not undermine the multi-hit framework proposed here, since genetic susceptibility, including the folate-pathway and mitochondrial vulnerabilities discussed in Section 3.1, is itself one of the proposed convergent pathways rather than a competing explanation. But it is a reminder that “convergent oxidative burden” as used throughout this paper describes a plausible, mechanistically grounded model rather than one that has been directly tested against confounding across all of its proposed component pathways simultaneously.

**Figure 2:**
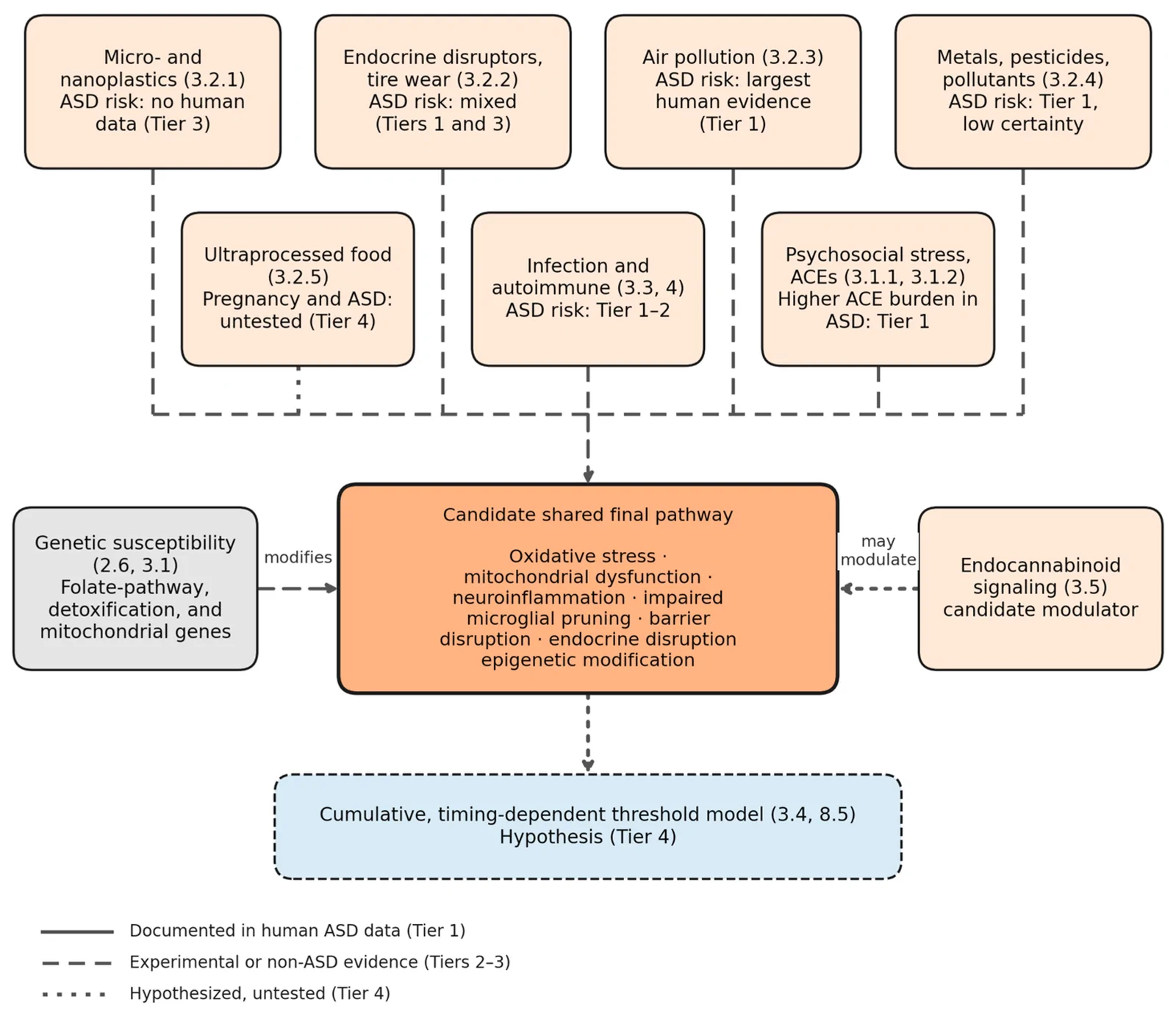
Seven distinct exposure classes across three domains (environmental chemicals, infection/autoimmune activity, and psychosocial stress/ACEs), each acting through a partially distinct sub-mechanism are proposed to converge on a shared final pathway of oxidative stress, mitochondrial dysfunction, neuroinflammation, barrier disruption, endocrine disruption, and epigenetic modification. Genetic susceptibility is proposed to modify vulnerability at this convergence point. See Figure 7 for how this shared burden interacts with buffering capacity and developmental timing to produce divergent clinical outcomes. Figure adapted and expanded from [4]. Line style indicates the evidence tier for each link: solid, documented in human ASD data (Tier 1); dashed, experimental or non-ASD evidence (Tiers 2–3); dotted, hypothesized and untested (Tier 4). Candidate modulators (genetic susceptibility, endocannabinoid signaling) are shown at the sides. Tier labels in the boxes refer to evidence on ASD risk or experimental effects; none of the links shown has been directly tested against regression, symptom severity, or diagnostic timing.

Figure 2 summarizes how these seven exposure sub-mechanisms, and genetic susceptibility, are proposed to converge on the shared final pathway described above.

### 3.5. The Endocannabinoid System as a Candidate Modulating Component

The endocannabinoid (eCB) system comprises the cannabinoid receptors CB1 and CB2, the lipid mediators anandamide (AEA) and 2-arachidonoylglycerol (2-AG), and the enzymes that synthesize and degrade them, such as fatty acid amide hydrolase (FAAH) and monoacylglycerol lipase (MAGL); a broader “endocannabinoidome” includes related lipid mediators and their receptors [85]. It is of interest here because it participates in processes that the preceding sections treat as routes to oxidative and inflammatory burden: stress and HPA-axis regulation (Section 3.1.1, Section 3.1.2 and Section 6), synaptic plasticity and neuroimmune signaling, gut barrier function and gut immunity, and interaction with the gut microbiome, which is itself shaped by dietary pattern (Section 3.2.5) [85]. It could therefore act as a modulating component of buffering capacity, or as an additional site where exposures converge, but this is a hypothesis (Tier 4).

Evidence in ASD is uneven. In humans, lower circulating anandamide and related lipid mediators have been reported in ASD children, with altered CB2 expression in peripheral blood cells (Tier 1, small studies) [85,86,87,88,89]. In rodent models (valproate-exposed, BTBR, and Fmr1-knockout), inhibitors of FAAH or MAGL and related agents improved social, repetitive, and anxiety-like behaviors, but effects were model- and context-dependent and some manipulations produced ADHD-like behaviors (Tier 3) [85]. Clinical studies of cannabis-derived products in ASD are mostly small, uncontrolled, or based on parent report, with heterogeneous products, and the randomized studies identified in one systematic review were single-dose imaging experiments that did not assess efficacy [86]; an expert review advises awaiting placebo-controlled trials [90]. Symptom improvement with a drug also does not show that the pathway is causal. No study has examined the eCB system in relation to regression, late diagnosis, oxidative burden, or the exposures discussed here (Tier 4). We include it as a candidate component of the buffering-capacity construct (Section 8.5), testable by measuring eCB mediators alongside oxidative and inflammatory markers in prospective cohorts.

## 4. Maternal Immune Activation, Autoimmune Disease, and Cerebral Folate Deficiency

This section summarizes a family of prenatal immune mechanisms, maternal immune activation, maternal autoimmune disease with autoantibody transfer, and cerebral folate deficiency via folate receptor alpha autoantibodies, that are distinct from, but mechanistically related to, the postnatal infection-triggered PANS/PANDAS pathway discussed in Section 3.3. Where PANS/PANDAS describes an abrupt postnatal event, these mechanisms operate substantially before birth and have independent support for a role in ASD risk and symptom severity generally. They are reviewed in detail in our earlier review (Sections 2, 4 and 5 of [8]), so only the points the present model depends on are given here, together with newer evidence published since that review. These prenatal mechanisms are discussed separately from the postnatal, infection-triggered syndromes of Section 3.3, because they involve a different developmental window and different evidence, and none has been related to the onset of regression.

### 4.1. Maternal Immune Activation: Cytokine Signaling and Fetal Brain Impact

Maternal immune activation (MIA), the pro-inflammatory maternal response to infection or systemic inflammation in pregnancy, is associated with increased ASD risk in human epidemiology, although the contribution of familial confounding remains debated (Section 3.4). Elevated IL-6, IL-17A, and TNF-α cross the placenta, activate fetal microglia, and disrupt neuronal migration, synaptic formation, and pruning. Human brain organoid models indicate that IL-6 and IL-17A alone are necessary and sufficient to induce ASD-like abnormalities, and blocking these cytokines prevents the social deficits otherwise produced by MIA in animals, which identifies the cytokine signal, not the infection itself, as the proximate mechanism in these experimental models (Tier 3). MIA effects on specific symptom domains have been traced to specific circuits, and, in animal models, its effects on the fetal brain are substantially mediated by microglial activation, oxidative stress, and mitochondrial dysfunction, the same convergent mechanisms implicated for the exposures in Section 3 of this manuscript (see Sections 2.1–2.4 of [8]). The natural-experiment evidence that infection itself, rather than a confounder, increases neuropsychiatric risk in the general pediatric population is described in Section 3.3.

A 2026 study strengthens the organoid evidence by using ‘cerebroids,’ ex vivo cultures made directly from human fetal dorsolateral prefrontal cortex that retain native tissue architecture, immune cells, vasculature, and extracellular matrix. IL-17A caused early cortical folding, a thicker cortex, and faster neurogenesis and neuronal maturation, resembling macrocephaly and increased prefrontal neuron number reported in some ASD individuals. IL-17A acted directly on neural stem cells to sustain NF-κB activation, a persistence that distinguished it from TNF-α, and NF-κB inhibition reversed the abnormalities, giving causal evidence, in this ex vivo human tissue system (Tier 3), for this step of the MIA-to-cortical-disruption pathway [91].

### 4.2. Maternal Autoimmune Disease and Autoantibody Transfer

Mothers with chronic autoimmune disease (for example systemic lupus erythematosus, Hashimoto’s thyroiditis, rheumatoid arthritis, and Sjögren’s syndrome) carry heightened systemic inflammation and, in a subset, brain-reactive autoantibodies that cross the placenta. Population-based studies in Taiwan, Europe, and North America consistently associate maternal autoimmune disease with roughly 30% higher odds of ASD in offspring, regardless of the specific diagnosis. Maternal IgG given during gestation produces ASD-like behavior in mice and non-human primates, and autoantibodies against fetal neuronal antigens define the candidate category “Maternal Autoantibody-Related (MAR) autism,” with reduced dendritic spines, altered cortical thickness, and disrupted connectivity: a proposed antibody-mediated route, supported mainly by animal models (Tier 3), to the same synaptic and connectivity disruption discussed throughout this paper (see Sections 4.1 and 4.2 of [8]).

### 4.3. Cerebral Folate Deficiency via Folate Receptor Alpha Autoantibodies

Autoantibodies against folate receptor alpha (FRα), the main transporter of folate across the blood–brain and placental barriers, can impair cerebral folate transport despite normal peripheral folate, producing cerebral folate deficiency (CFD). Roughly 33–70% of ASD children test positive for folate receptor autoantibodies (FRAAs), compared with about 5–10% of neurotypical children, among the most replicated biomarker differences in the ASD literature [8,92]. CFD connects to the oxidative-stress framework by a proposed route that has not been tested in ASD: impaired folate metabolism raises homocysteine, which independently increases intracellular oxidative stress, and folate is needed for myelination, monoamine synthesis, and GABA/glutamate balance. Folinic acid (leucovorin), which bypasses the blocked receptor through the reduced folate carrier, improved communication and adaptive functioning in FRAA-positive children in several randomized, placebo-controlled trials, with benefit greatest when treatment began at ages 2 to 3 and declining sharply after age 7, a critical-period pattern analogous to the developmental-window framing used here [8,93]. FRAA prevalence, neurodevelopmental consequences, and folinic acid treatment are reviewed in Sections 5.2–5.4 of [8].

### 4.4. Synthesis: Autoimmune Mechanisms Within This Paper’s Oxidative-Stress Framework

The three mechanisms converge with the chemical, infectious, and psychosocial pathways of Section 3 on shared downstream targets: microglial activation and impaired synaptic pruning, oxidative and mitochondrial stress, and, for CFD, a proposed homocysteine-mediated oxidative mechanism (compare Section 6 and Figure 1 of [8]). They are also the mechanisms most directly connected to the gut-microbiome and folate pathway developed in Section 5, since microbially derived folate can modulate host folate receptor expression, raising the untested possibility that microbial composition could shift folate receptor availability independent of autoantibody status. Whether pollutant-driven microbiome disruption (Section 3.2) or family stress (Section 6) raises the likelihood of FRAA production or MIA-triggering infection is untested directly and is a research priority [8,94]. Evidence status: maternal immune activation and maternal autoimmune disease are supported by human epidemiology (Tier 1), although a large population-based analysis specifically examined whether familial confounding could explain the MIA–ASD association (Section 3.4), and mechanistic support comes from animal and organoid models (Tier 3). FRAA positivity is a replicated biomarker finding in ASD (Tier 1), supported by small randomized trials of folinic acid in FRAA-positive children, but the proposed route from cerebral folate deficiency to oxidative burden through homocysteine, and any link to regression, remain Tier 4.

**Figure 3:**
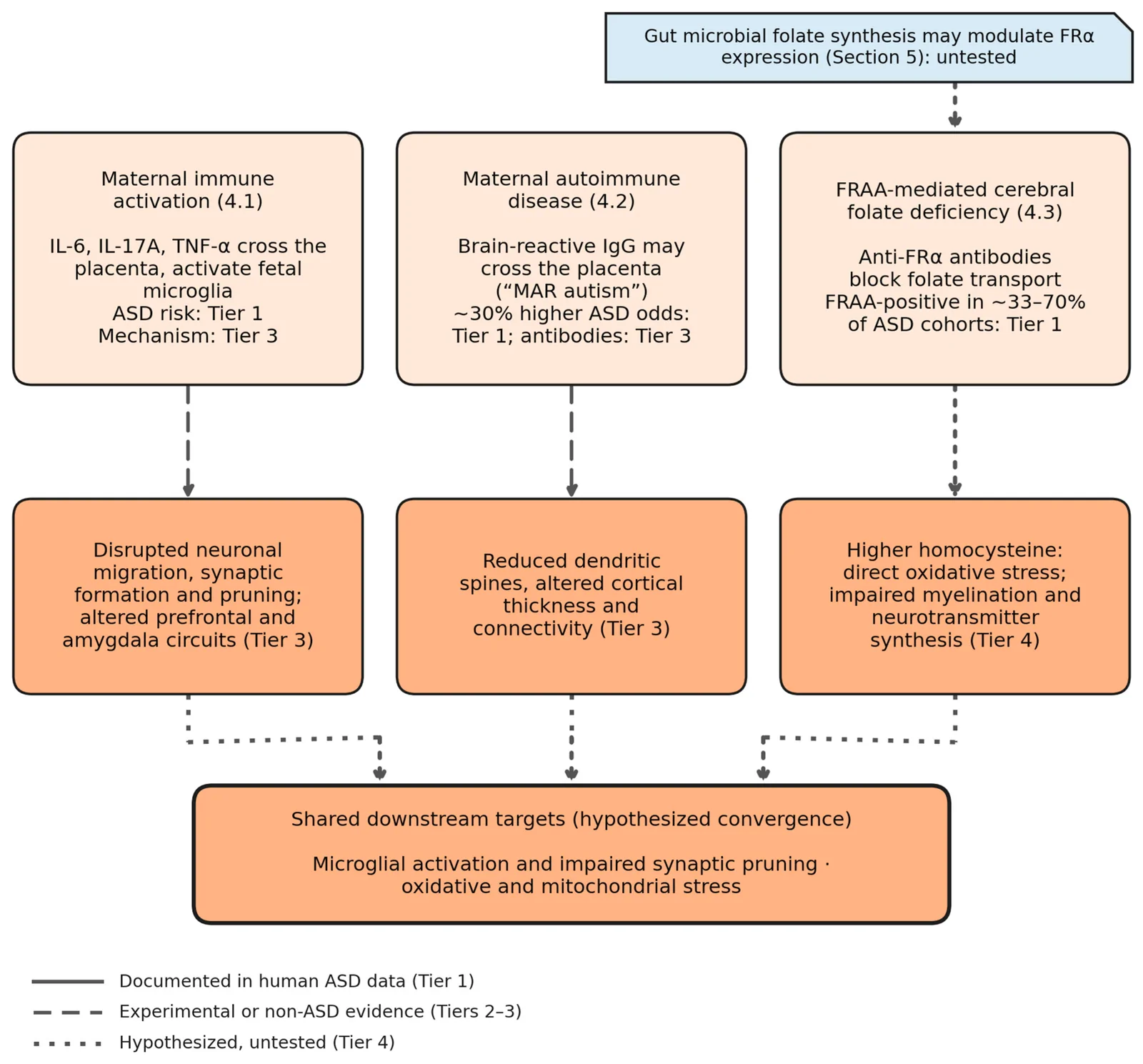
Three mechanistically distinct prenatal immune pathways, maternal immune activation, maternal autoimmune disease with autoantibody transfer, and folate receptor alpha autoantibody-mediated cerebral folate deficiency, are proposed to converge on shared downstream targets of microglial activation, impaired synaptic pruning, and oxidative/mitochondrial stress. The FRAA route is proposed to carry an additional oxidative mechanism via elevated homocysteine (Tier 4). Line style indicates the evidence tier for each link: solid, documented in human ASD data (Tier 1); dashed, experimental or non-ASD evidence (Tiers 2–3); dotted, hypothesized and untested (Tier 4).

Figure 3 summarizes how these three prenatal immune mechanisms converge on shared downstream targets.

## 5. The Gut Microbiome as a Mediating Pathway

### 5.1. Microbiome, Oxidative Stress, and One-Carbon Metabolism

The gut microbiome influences host oxidative stress both directly, by generating and metabolizing reactive oxygen species, and indirectly, by shaping systemic inflammatory tone through the gut–immune interface. Specific microbial taxa also contribute to the synthesis and bioavailability of B vitamins that underpin antioxidant and methylation capacity, making the microbiome a plausible mediating layer between environmental exposure (Section 3) and downstream neurodevelopmental vulnerability [94]. This role is treated here only in summary; the microbiome as a modifier of folate/vitamin B availability and neuroinflammation is developed in Section 6 of [9].

Two features of this pathway matter for the present model. First, the capacity for folate production is unevenly distributed: a survey of gastrointestinal reference genomes found that a minority carry the complete de novo folate synthesis pathway and a larger group can synthesize folate given dietary or cross-fed para-aminobenzoic acid, with Bifidobacterium and Lactobacillus standing out as producers and microbially derived folate able to modulate host folate-receptor expression. This raises the untested possibility that microbial composition could shift folate receptor availability independent of autoantibody status [94]. Second, the relationship is bidirectional: B9/B12 intake and diet quality shape microbial richness and the abundance of these same genera, and Western-diet-pattern dysbiosis can increase intestinal permeability and lipopolysaccharide translocation, activating TLR4 on intestinal immune cells and microglia and driving neuroinflammation and altered synaptic pruning, the process implicated for microplastics (Section 3.2.1) and maternal immune activation (Section 4.1). Gut-derived short-chain fatty acids such as propionate add an ASD-specific signal [95].

### 5.2. B9/B12 and Methylation Capacity

Folate (vitamin B9) and vitamin B12 are essential cofactors in the one-carbon metabolic cycle, which governs methylation reactions throughout the body, including in processes relevant to neurodevelopment and to the synthesis of glutathione, the body’s primary intracellular antioxidant. Reduced bioavailability of either vitamin, whether from dietary insufficiency, gut absorption impairment, or microbiome composition that disfavors their synthesis or supports their consumption by competing microbes, could in principle reduce methylation and antioxidant capacity together, a biochemical route from microbiome disruption to oxidative vulnerability that is supported by experimental and nutritional work but has not been demonstrated in ASD cohorts; folate or vitamin B12 deficiency has not been shown to result from microbiome changes in autistic children, and we do not assume that it does [94,95].

### 5.3. Genetic Susceptibility Modifiers

Host genotype shapes both how much folate reaches the brain and how much oxidative stress a given exposure produces, so the microbiome–folate pathway does not act on a uniform substrate. Two genetic domains are most relevant to this framework: folate-transport and one-carbon genes, notably *MTHFR* C677T and *SLC19A1* rs1051266, which reduce the metabolic reserve available to buffer one-carbon disruption (including that attributed here to plastic-associated endocrine disruptors, Section 3.2.2); and xenobiotic-detoxification genes, notably *PON1* and the glutathione S-transferase genes *GSTM1* and *GSTT1*, whose variants appear to modify risk when chemical exposure is present. These domains are discussed in [9]. None of the genotype studies has yet been tested directly against the micro/nanoplastic or endocrine-disrupting exposures described in Section 3.2, which is a concrete, near-term priority for testing the cumulative-threshold model: the same exposure dose should be expected to produce different regression or diagnosis-timing outcomes depending on a child’s folate-transport and detoxification genotype.

### 5.4. Microbiome Evidence in ASD, Including Regression

At least one study has directly examined the question of a regression-specific microbiome signature: a comparison of fecal microbiota in ASD children, who are with versus without documented mental regression, motivated by the premise that gut microbial shifts could produce clinically meaningful behavioral change through toxin production, altered fermentation byproducts, and immune/metabolic effects. The authors are candid that evidence specific to regression remains scarce, this is a small, early literature study rather than a settled one, and should be read as suggestive rather than confirmatory [96].

The strongest available test of this section’s core premise, that early gut microbial composition predicts later regression or diagnosis, comes from the MARBLES prospective cohort, which analyzed fecal samples collected between 0 and 7 months of age via 16S rRNA sequencing against clinical classification at around 36 months. Neither overall community diversity within or between samples nor the abundance of any individual taxon distinguished infants who were later diagnosed as ASD from those who developed typically, a well-powered, prospectively designed null result in a cohort where a genuine effect should be easiest to detect. This tempers, without ruling out, the mechanistic case built from the bacterial folate-synthesis and diet-microbiome literature above; it is possible that microbiome composition affects risk too subtly, too heterogeneously across individuals, or too early, via in utero or maternal-microbiome influences that predate this sampling window, for this particular design to detect [97].

Two further studies complicate rather than confirm the mediation hypothesis. In adults evaluated for metabolic dysfunction-associated steatotic liver disease, fecal microplastic burden correlated with an increase, not a decrease, in fecal Bifidobacterium, a principal folate-producing genus, alongside reduced Lachnospiraceae [98]. This is the only study identified that measured microplastic burden and gut microbial shifts together in living humans, and it suggests that microplastic–microbiome interactions do not run in one uniform direction. In addition, a 2026 pilot randomized trial of a multi-strain Bifidobacterium/Lactobacillus probiotic in young autistic children raised fecal Bifidobacteriaceae abundance relative to placebo without a significant change in short-chain fatty acids or parent-reported behavior [99]. Read together with the MARBLES null result, these findings indicate that microbiome mediation is plausible and partly testable with existing methods but has not been confirmed at either the risk-prediction or the intervention level.

Beyond regression-specific data, the broader ASD microbiome literature is heterogeneous. Reported ASD-associated profiles include a lower Bacteroidetes-to-Firmicutes ratio, more Proteobacteria, and altered short-chain fatty acid-producing taxa, but findings differ between studies and, in animal models, even within the same model, and sex, genetic background, and environment further modify them [85]. Case–control comparisons are also open to confounding and reverse causation: ASD children often have selective eating and restricted diets, gastrointestinal symptoms such as constipation, and exposure to medications and supplements, each of which alters the microbiome, so dysbiosis may follow from diet, symptoms, or treatment rather than precede or cause the condition. Technical and geographical differences are a further source of discordance: study populations and laboratory and computational pipelines differ between datasets, and an analysis of an original dataset plus seven public case–control datasets used a statistical correction for these batch effects and still identified a distinct microbiota profile in ASD, with *Bacteroides*, *Faecalibacterium*, *Gemmiger*, *Blautia*, *Bifidobacterium*, and *Phocaeicola* as the most robust markers [100]. A sibling-controlled machine-learning analysis selected 26 taxa that classified ASD with an average AUC of about 0.82 in the discovery data and about 0.74–0.75 in two independent cohorts with unrelated controls, although the authors note that specific taxa rarely agree across studies and that diet, lifestyle, sex, geography, and sequence-processing choices all influence results [101]. Both are case–control classification studies (Tier 1): they show that ASD status and microbiome composition are associated, not that the microbiome contributes to onset, and the second discusses a study that attributed such associations to autism-related dietary preferences [101].

### 5.5. Proposed Causal Chain

Synthesizing Section 3, Section 4 and Section 5, we propose the following hypothesized chain (not a demonstrated causal pathway): environmental exposure (chemical, dietary, or infectious/autoimmune) disrupts gut microbiome composition; microbiome disruption reduces bioavailability of B9/B12 and related cofactors; reduced cofactor availability impairs both methylation and glutathione-dependent antioxidant capacity; reduced antioxidant capacity lowers the threshold at which a given exposure, or a subsequent infection, or a period of psychosocial stress (Section 6), precipitates visible regression or symptom emergence. The links differ in support: effects of exposure on the microbiome come mainly from animal models (Tier 3); microbial folate synthesis from genome surveys and experimental work (Tier 3); the effect of reduced cofactors on methylation and glutathione from general biochemistry; and the final link to regression or symptom emergence is Tier 4. The chain as a whole, including its first link, has not been tested (see the MARBLES null result, Section 5.4).

**Figure 4:**
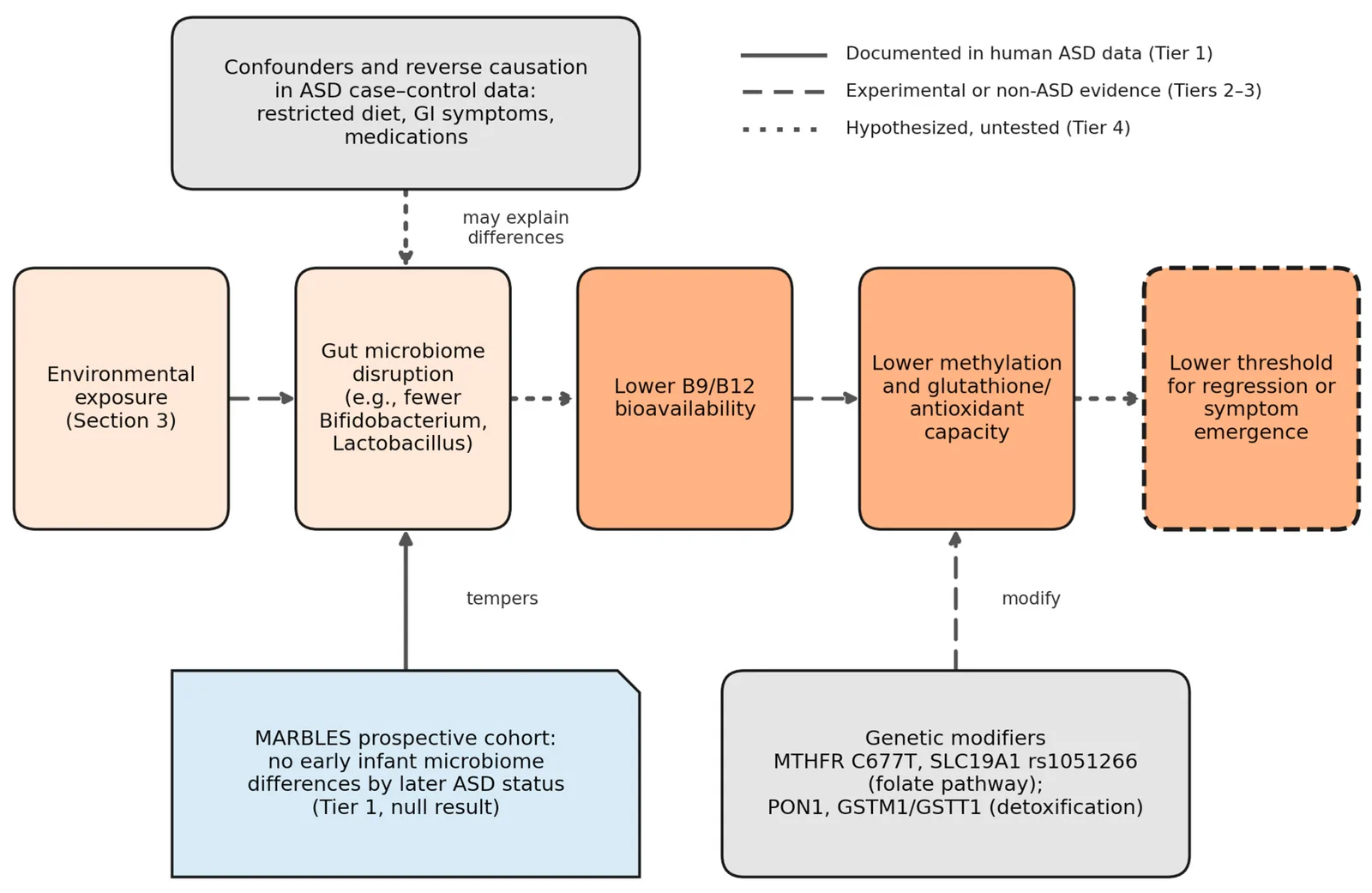
Our proposed causal chain linking environmental exposure to regression or symptom-emergence risk via gut microbiome disruption, reduced B9/B12 bioavailability, and impaired methylation/antioxidant capacity. Folate-pathway and xenobiotic-detoxification genetics modify specific links in the chain. Individual links rest mainly on experimental, genomic, or general biochemical evidence (Tiers 3–4), not on human ASD data; the chain as a whole, including the first link, has not been confirmed (the MARBLES cohort found no significant microbiome differences by later ASD status). Line style indicates the evidence tier for each link: solid, documented in human ASD data (Tier 1); dashed, experimental or non-ASD evidence (Tiers 2–3); dotted, hypothesized and untested (Tier 4).

Figure 4 summarizes this proposed causal chain and the genetic factors that modify specific links in it.

## 6. Psychosocial Stress and Cortisol

### 6.1. The HPA Axis and Oxidative Stress: A Bidirectional Relationship

The hypothalamic–pituitary–adrenal (HPA) axis and its main effector hormone, cortisol, are reciprocally linked to oxidative stress, a relationship that is well described in general physiology but has not been tested in ASD. Chronic HPA activation elevates markers of oxidative damage, while oxidative stress itself can dysregulate HPA reactivity, potentially creating a self-reinforcing cycle under conditions of sustained stress exposure. This bidirectionality is relevant to the model proposed here because it suggests psychosocial stress need not act only as an independent contributor to regression risk, it may also amplify the oxidative consequences of the environmental exposures discussed in Section 3 [6].

The closest large-scale human evidence comes from a Danish nationwide cohort of over 1 million infants, which compared children prenatally exposed to systemic (pharmacological) glucocorticoids, prescribed either for risk of preterm delivery or for maternal autoimmune/inflammatory disease, against unexposed children born to mothers with the same underlying condition, a design intended to reduce confounding by indication relative to a general-population comparison. Exposed offspring had a higher adjusted 15-year risk of ASD in both the preterm-delivery-risk group (6.6% vs. 4.3%; RR 1.5, 95% CI 1.2–1.9) and the autoimmune/inflammatory-disease group (4.8% vs. 3.8%; RR 1.3, 95% CI 1.1–1.5), alongside elevated ADHD and mood/anxiety/stress-related disorder risk, with findings holding up in a sibling-matched sensitivity analysis [102]. The authors point to fetal HPA-axis programming and altered CNS structural development following excess glucocorticoid exposure during specific gestational windows as the likely mechanism, the same pathway invoked here for endogenous, stress-driven cortisol elevation. While exogenous pharmacological glucocorticoids and chronic maternal psychological stress acting through endogenous cortisol are not identical exposures, and this study cannot speak directly to the latter, the shared mechanism, excess glucocorticoid signaling during a gestational critical window reprogramming fetal HPA-axis and CNS development, offers the closest large-scale human analogue currently available for the maternal-stress pathway we propose, and supports treating maternal stress-axis dysregulation as a coincident-timing risk factor structurally analogous to the chemical exposures discussed in Section 3.2, rather than as a qualitatively different mechanism.

### 6.2. Family and Psychosocial Stress Evidence in ASD

There is active, if still developing, research specifically addressing psychosocial stress in autistic populations. Kern and colleagues have developed the Childhood Adversity and Social Stress Questionnaire (CASSQ), an instrument measuring adverse and traumatic experiences and trauma symptoms in autistic youth; notably, its symptom subscale explicitly includes regression of skills and increased self-injurious behavior as trauma-related outcomes, treating regression itself as a measurable consequence of stress exposure rather than solely a neurodevelopmental event. More broadly, ASD children are documented to experience adverse childhood experiences (ACEs) at higher rates than non-ASD children, particularly in lower-income families, and autistic adults report significantly more adverse events across the lifespan than neurotypical adults. Supporting biological plausibility, animal research shows early-life stress increasing susceptibility to autoimmune neuroinflammatory disease and worsening its severity, a finding relevant to the infection/autoimmune pathway discussed in Section 3.3 [103,104,105,106,107,108].

### 6.3. Proposed Synergy with Environmental Exposure

We hypothesize that family and psychosocial stress could function as an additive, and potentially synergistic, oxidative load layered on top of environmental chemical and infectious exposure, rather than as a competing explanation for regression or late symptom emergence. Under the bidirectional HPA–oxidative stress relationship described in Section 6.1, a child already carrying elevated oxidative burden from chemical exposure, dietary factors, or microbiome disruption (Section 3, Section 4 and Section 5) may be more vulnerable to psychosocial stress precipitating visible regression than a child without that baseline burden, and conversely, chronic family stress may lower the threshold at which a subsequent infection or exposure precipitates decline. This remains a hypothesis: the stress–regression link has not been tested (Section 6.4), and the direction of effect may in part run the other way, because raising an autistic child with high support needs, or an unrecognized difficulty, is itself stressful for families. Nothing in this section implies that parents or families cause autism or regression; psychosocial stress is considered as one of several environmental loads, many of which reflect socioeconomic and structural conditions that families do not control.

### 6.4. Caveat

It should be stated plainly that no existing study directly links ACE burden to regression episodes in already-diagnosed autistic children in the way the PANS/PANDAS literature links infection to regression. The CASSQ work establishes that regression is measured as a trauma-related symptom in principle, but a direct empirical test of stress burden predicting regression onset, analogous to the infection-triggered PANS/PANDAS studies, has not, to our knowledge, been conducted.

**Figure 5:**
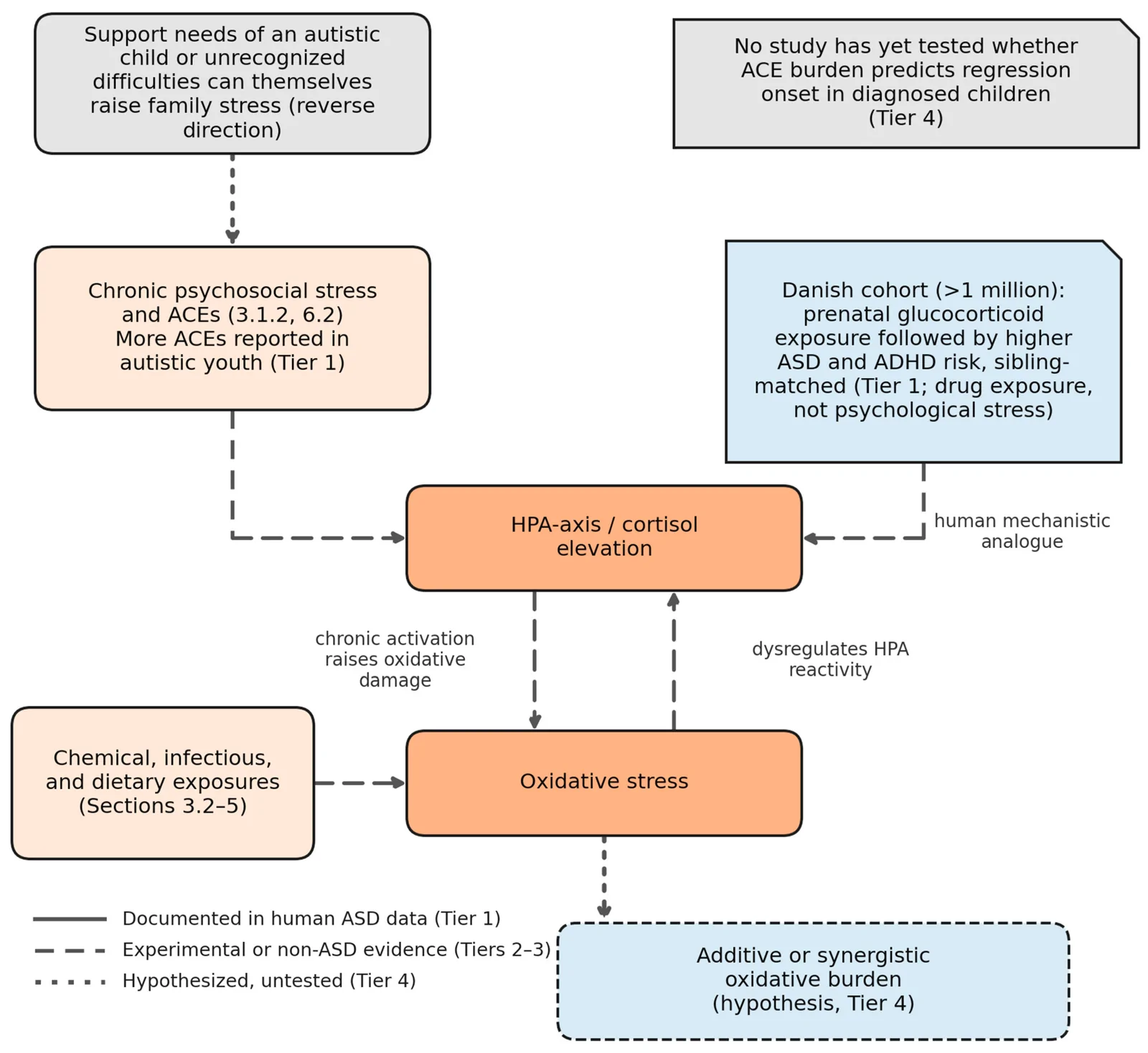
Chronic psychosocial stress and ACEs elevate HPA-axis/cortisol activity, which is proposed to both drive and be dysregulated by oxidative stress, a bidirectional relationship described mainly in experimental and non-ASD work; the closest large-scale human support is a Danish cohort linking prenatal glucocorticoid exposure (a drug exposure, not psychological stress) to ASD risk. This paper proposes the resulting oxidative burden is additive or synergistic with, rather than competing with, the chemical and infectious exposures addressed in Section 3, Section 4 and Section 5. No study has yet directly tested ACE burden against regression onset in diagnosed children. Line style indicates the evidence tier for each link: solid, documented in human ASD data (Tier 1); dashed, experimental or non-ASD evidence (Tiers 2–3); dotted, hypothesized and untested (Tier 4).

Figure 5 summarizes this bidirectional HPA–oxidative stress relationship and its proposed additive effect on the environmental burden discussed elsewhere in this paper.

## 7. Sex Disparity in Diagnostic Timing, Reframed

### 7.1. The Data

Recent large-sample data provide a clear picture of the sex disparity in diagnostic timing. Epic Research’s 2024 analysis of patient records found that the median age at diagnosis for boys fell to about 5 years (from 7 in 2015), whereas for girls it stayed at roughly 8 years, essentially unchanged over the period. In 2024, 44% of boys but 34% of girls received a diagnosis before age 5, and almost one in four girls (versus 12% of boys) were first diagnosed in adulthood, at age 19 or older. The average age at diagnosis for women increased over the past decade, from 11.7 years in 2015 to 13.1 years in 2024, while men’s average dropped, indicating the sex gap in diagnostic timing is widening rather than closing on this measure, even as median ages fall overall [109].

Registry data from Sweden covering 2.76 million people born from 1985 to 2020 showed a male-to-female diagnosis ratio of about 3:1 through age 10 that then fell steadily with age; diagnoses peaked at ages 10–14 in males but at ages 15–19 in females, and by age 20 the cumulative ratio had narrowed to about 1.2:1 as of 2022, with projections of near parity by age 20 in more recent birth cohorts. CDC ADDM surveillance data show a parallel, if less pronounced, trend in childhood prevalence ratios, narrowing from 4.2:1 in 2018 to 3.4:1 in 2022 among 8-year-olds, though researchers caution this narrowing may not solely reflect improved identification of girls [110,111].

Prevalence ratios by sex also bear on this question. Male-to-female ratios of about 3:1 to 5:1 are reported depending on the source [27,112,113], and the registry and surveillance data above show the ratio narrowing with age. Two non-exclusive classes of explanation appear in the genetic literature: male-biased risk factors, such as sex steroid hormone signaling and its prenatal programming of brain development, and a female protective effect in which females tolerate a larger burden of risk variants before an ASD phenotype appears; autistic females tend to carry more rare de novo variants and a higher polygenic risk [112,113].

Experimental work on UBE3A, a gene on chromosome 15q11–13 whose duplication or triplication accounts for about 1–2% of autism, illustrates how sex-differential biology could act on ASD-relevant phenotypes. In mice with extra copies of *Ube3a*, effects on brain connectivity, behavior, and gene expression were sex-biased, including expression of X-linked and steroid-hormone-influenced genes [112]. In a second Ube3a-overexpressing model, males showed greater deficits in social communication, memory, and pain sensitivity; UBE3A-mediated degradation lowered androgen receptor levels in both sexes, but only males showed dysregulated androgen receptor target genes, and restoring androgen receptor levels rescued deficits in males without affecting females [113]. These are animal models of one genetic form of ASD (Tier 3) and address the male excess in prevalence, not the timing of diagnosis; they support the plausibility, but not the truth, of sex-linked differences in biological buffering (Section 7.3). ASD females are also reported to show more internalizing symptoms and males more externalizing symptoms [113], a difference in presentation that bears on recognition (Section 7.2).

### 7.2. The Dominant Explanation: Masking and Diagnostic Criteria

The most consistently supported explanation for this disparity is that autism in girls and women often presents differently than the male-typical profile on which diagnostic criteria and clinician training have historically been based, including greater social “masking” capacity and special interests that are less overtly atypical. This leads to years of alternative diagnoses (anxiety, depression, borderline personality disorder, OCD) before ASD is correctly identified, and to lower first-evaluation accuracy for women even when they are referred. This explanation is well-supported across multiple independent data sources and is not displaced by anything proposed in this paper [114,115].

### 7.3. A Complementary Hypothesis: Sex Differences in Oxidative Buffering

We offer, as a hypothesis for future testing and not as an established mechanism, that sex differences in oxidative-stress buffering capacity could contribute to the later or subtler presentation seen on average in females, in addition to clinician masking. The rationale is limited: estrogen has antioxidant properties in experimental systems, and HPA-axis and cortisol reactivity differ by sex, but we are not aware of direct evidence that these differences affect the timing of ASD symptom emergence or diagnosis (Tier 3 and Tier 4). Under the threshold model (Section 8.5), if average female buffering were greater, the same cumulative burden would take longer to cross a functional threshold, which would appear statistically as later diagnosis and a larger share of adult diagnoses. This hypothesis does not require rejecting the masking explanation, but it carries an important qualification: it concerns the timing of symptom expression and recognition and does not imply that autism in girls and women begins biologically later. Distinguishing the accounts requires sex-stratified prospective data that include measures of camouflaging, as specified in Section 8.5 (prediction 5).

### 7.4. The Narrowing Ratio as Indirect, Non-Definitive Support

The Swedish registry result that the male-to-female ratio of diagnoses closes toward parity (about 1:1) by age 20 is consistent with, though does not prove, a cumulative-threshold model: if a comparable underlying rate of autism exists in both sexes but girls take longer, on average, to cross a symptom-severity or recognition threshold, later-life registry data would be expected to show exactly this convergence pattern. This observation is, however, equally consistent with a pure masking/recognition account and the current data cannot distinguish between the two explanations. This is flagged as a direction for future research (Section 9) rather than as support already secured for the oxidative-buffering hypothesis [111].

**Figure 6:**
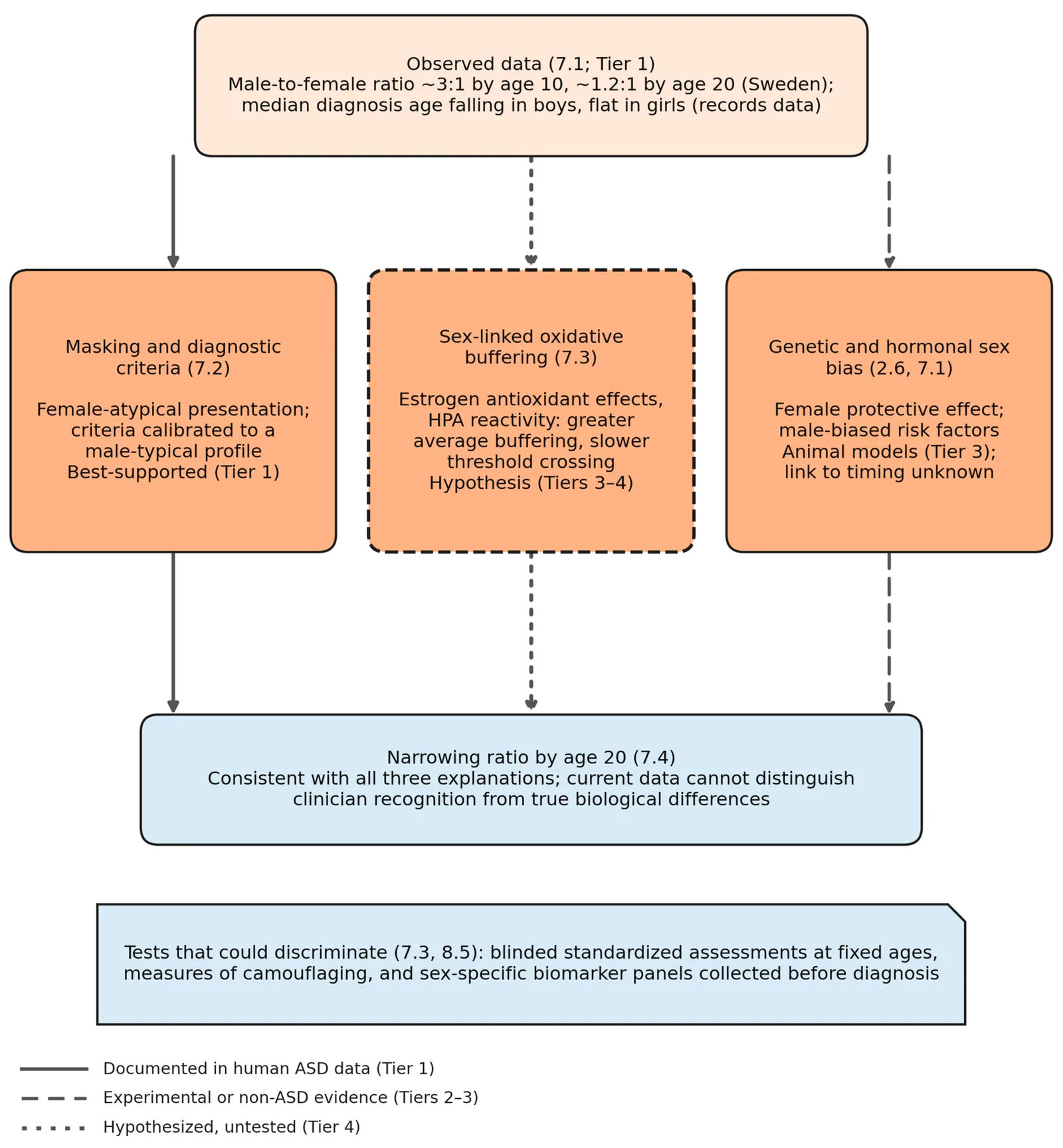
The well-documented narrowing of the male-to-female autism diagnosis ratio with age is consistent with several non-exclusive explanations: the well-supported masking/diagnostic-criteria account, genetic or hormonal sex bias (animal models; link to timing unknown), and this paper’s proposed oxidative-buffering hypothesis. Current data cannot distinguish clinician recognition effects from genuine physiological buffering differences Line style indicates the evidence tier for each link: solid, documented in human ASD data (Tier 1); dashed, experimental or non-ASD evidence (Tiers 2–3); dotted, hypothesized and untested (Tier 4).

Figure 6 summarizes these explanations (masking, oxidative buffering, and genetic or hormonal sex bias) and the narrowing-ratio data pattern with which all are consistent.

## 8. Integrated Model

Synthesizing Section 3, Section 4, Section 5, Section 6 and Section 7, we propose the following hypothesized integrated model. Environmental exposures, chemical, dietary, and infectious/autoimmune, combine with family and psychosocial stress to elevate cumulative oxidative and inflammatory burden. This burden is mediated, in part, by gut microbiome disruption, which reduces bioavailability of B9/B12 and thereby impairs both methylation and glutathione-dependent antioxidant capacity. The resulting oxidative burden interacts with individual buffering capacity, shaped by genetics, baseline exposure history, and possibly sex-linked physiological differences in antioxidant and HPA-axis function, to determine when, and whether, a visible behavioral threshold is crossed. Depending on the developmental window in which that threshold is crossed, and depending on whether the crossing is abrupt (as in an infection-triggered PANS/PANDAS event) or gradual (as in the Elias & Lord “gained diagnosis” trajectory [18]), the clinical presentation may take the form of early toddler regression, late regression after a period of stability, or late diagnosis without a prior documented symptom history. Each link in this model is a hypothesis; the evidence tier for each is given in Table 2, and the human ASD evidence concerns ASD risk rather than regression, symptom severity, or diagnostic timing, except where Table 2 states otherwise.

### 8.1. Multiple Convergent Paths to a Shared Burden

The preceding sections describe several superficially distinct studies, environmental chemical exposure, infection and autoimmune activity, psychosocial stress and ACEs, gut microbiome and folate-B12 status, each independently associated with autism risk. Our central hypothesis is that these are not competing explanations but convergent inputs to a single shared burden: cumulative oxidative and inflammatory stress, modified by individual genetic susceptibility (Section 3.1) and buffered by individual antioxidant reserve and HPA-axis reactivity (Section 6 and Section 7.3). No single exposure class is proposed as necessary or sufficient on its own; a child could plausibly reach the same functional burden through chemical exposure alone, through chronic psychosocial stress alone, or, more commonly in this model, through some combination of moderate exposures across several domains that no single-exposure study would detect as significant in isolation.

### 8.2. Critical-Period Timing and the Divergence of Clinical Presentation

What determines the clinical presentation, in this model, is not only the size of the cumulative burden but the developmental timing at which it crosses, if it ever crosses, a functional threshold, together with whether that crossing is abrupt or gradual. Five broad patterns follow from this framework, and each of the five clinical patterns has been documented empirically (Section 2), but their attribution to the timing of a threshold crossing is our hypothesis and has not been tested. First, if buffering capacity is sufficient relative to lifetime burden, the threshold may never be crossed and no ASD phenotype results, the implicit comparison case against which the other four patterns are defined. Second, if the threshold is crossed prenatally or in very early postnatal life, most typically through high chemical or infectious burden during the third-trimester and early-postnatal windows repeatedly identified in Section 3.2 as periods of particular vulnerability for ASD risk, the result is early-onset ASD without a regression history, the classical and most-studied presentation. Third, if early burden is substantial but sub-threshold, a second, discrete hit, an infection meeting PANS/PANDAS criteria (Section 3.3), an acute household stressor, or a further chemical exposure, occurring around 12–24 months could push cumulative burden over threshold abruptly, producing the early (toddler) regression pattern described in Section 2.1. Fourth, if burden remains sub-threshold through early childhood but a later hit, chronic ACE exposure (Section 3.1.2), a later infection, or accumulating chemical burden, occurs after a period of apparent developmental stability, the result would be late regression, the pattern whose mean onset around age 13 (Section 2.2) falls within the adolescent window of heightened sensitivity discussed in Section 3.1, which is compatible with, but does not demonstrate, a chronic ACE/HPA-axis contribution. Fifth, if burden accumulates gradually across childhood without ever producing an abrupt crossing, and buffering capacity is comparatively high, symptoms may emerge slowly enough to resemble the Elias and Lord “gained diagnosis” trajectory described in Section 2.3, producing late diagnosis without a documented childhood symptom history rather than a discrete regression event.

### 8.3. Compensation, Masking, and the Sex Disparity

Buffering capacity does double duty in this framework, and the distinction matters clinically. Beyond preventing a crossing altogether (Section 8.2), buffering may slow it, delay it, or produce a milder, more compensable phenotype that is harder for caregivers and clinicians to recognize as autism, particularly against diagnostic criteria and clinician training historically calibrated to a male-typical presentation (Section 7.2). Our proposed sex-linked buffering hypothesis (Section 7.3), that estrogen’s antioxidant properties and/or sex differences in HPA-axis reactivity provide somewhat greater average buffering capacity in females, offers one candidate explanation, complementary to and not competing with the masking/diagnostic-criteria explanation, for why the same cumulative burden might on average take longer to produce a recognizable, diagnosable presentation in girls than in boys. Under this combined view, the well-documented sex disparity in diagnostic timing (Section 7.1) could reflect some mixture of clinicians failing to recognize a fully present phenotype (pure masking) and female physiology genuinely delaying, on average, how long it takes a given cumulative burden to cross a functional threshold (buffering); the two mechanisms are not mutually exclusive and current data cannot cleanly separate their relative contributions, a limitation already noted in Section 7.4. It remains secondary to the well-supported masking account and concerns the timing of expression and recognition, not a later biological onset (Section 7.3).

### 8.4. Schematic Model

**Figure 7:**
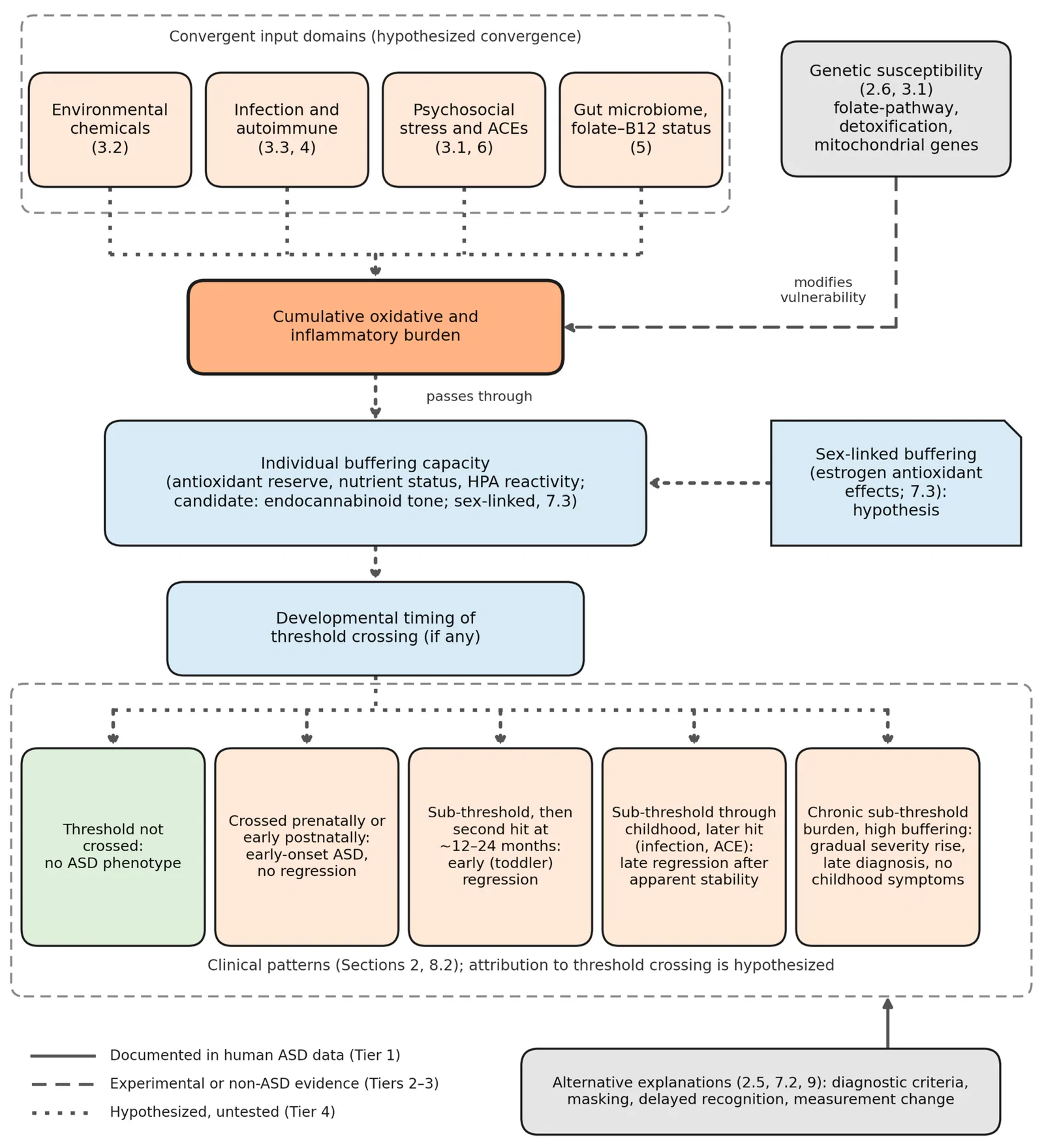
Multiple convergent input domains combine into a cumulative oxidative/inflammatory burden, modified by genetic susceptibility and passed through individual (including sex-linked) buffering capacity. The developmental timing of any resulting threshold crossing, and whether that crossing is abrupt or gradual, is proposed to determine which clinical pattern, if any, results: no ASD phenotype, early-onset ASD, early (toddler) regression, late regression after apparent stability, or a gradual late-diagnosis trajectory without a documented childhood symptom history. Line style indicates the evidence tier for each link: solid, documented in human ASD data (Tier 1); dashed, experimental or non-ASD evidence (Tiers 2–3); dotted, hypothesized and untested (Tier 4).

Figure 7 summarizes this model schematically: convergent input domains combine into a cumulative oxidative/inflammatory burden, modified by genetic susceptibility and passed through individual (including sex-linked) buffering capacity, with the developmental timing of any resulting threshold crossing proposed to determine which of the clinical patterns described in Section 8.2, if any, results.

### 8.5. Operational Definitions, Testable Predictions, and Falsifiability

To be more than a metaphor, the model needs terms that can be measured. We use the following working definitions, which are proposals for testing rather than validated constructs. Cumulative burden is the time-integrated net pro-oxidant and pro-inflammatory load on the developing brain, estimated from exposure biomonitoring or validated exposure models together with biomarkers of oxidative damage and inflammation (for example markers of oxidative DNA and lipid damage, protein oxidation, hs-CRP, and cytokines) and, where available, cord-blood or newborn-spot metabolomic profiles. Buffering capacity is the ability to neutralize or repair that load, approximated by glutathione redox status, antioxidant enzyme activity, folate and vitamin B12 status (including bioactive folate forms), relevant genotypes (detoxification, folate-pathway, and mitochondrial genes), FRAA status, microbiome functional capacity, HPA-axis reactivity, and, as a candidate, endocannabinoid tone. A threshold is a point on the burden-versus-buffering relationship beyond which the risk of regression or symptom emergence rises nonlinearly; empirically, it would appear as a better fit of a piecewise or sigmoidal burden–response function than of a linear one. Burden and buffering are continuous and measured with error, so the threshold is a statistical construct, not a single biochemical value.

In prospective cohorts that sample before onset (for example infant-sibling or birth cohorts), the model predicts the following: (1) children who later regress, or whose symptoms later increase, show steeper pre-onset rises in burden markers or falls in buffering markers than matched children without regression, including children with early-onset ASD without regression; (2) exposure and buffering interact, so that the same exposure carries a higher risk of regression in children with lower buffering (for example certain genotypes, low bioactive folate, or FRAA positivity) than expected from additive effects; (3) the burden–outcome relationship is better described by a nonlinear threshold function than by a linear dose–response; (4) discrete hits (infection, acute stress, a new exposure) are more likely to precipitate regression when baseline burden is high or buffering low; and (5) sex differences in buffering markers, measured before diagnosis, predict later symptom emergence independently of camouflaging and referral patterns. Observations that would count against the model include no pre-onset difference in burden or buffering markers, markers that differ only after onset, no exposure × buffering interaction in adequately powered mixture analyses, linear burden–response relationships that fit as well as threshold models, and sex differences that disappear once camouflaging and ascertainment are modeled.

Several design issues must be addressed. Oxidative-stress markers are nonspecific and are affected by diet, medication, sleep, gastrointestinal symptoms, and illness, so correlations measured after onset cannot distinguish cause from consequence; this is why prospective, pre-onset sampling is required, and, where genetic instruments exist, Mendelian randomization could help separate cause from consequence. Additive, interactive, and threshold-like effects can be separated only with mixture methods and interaction terms in cohorts that measure several exposure classes and genotypes together, which few currently do. Regression itself is usually defined retrospectively, so prospective standardized developmental assessment is needed. Finally, support for the model would not show that oxidative stress is the final common pathway for any individual child; it would show that burden, buffering, and timing add predictive information about trajectories beyond established explanations.

### 8.6. Evidence Status of the Key Links

Table 2 summarizes the tier, best available evidence, main limitations, and status of each link in the proposed model, with the section in which each is discussed. It is intended to make clear which links rest on human ASD data, which on other human or experimental data, and which are hypotheses. Key supporting studies are now cited in the “Best available evidence” and “Main limitations” columns, with single-study findings cited individually so that readers can verify them; statements in the table without a citation are not supported by a specific study in this review.

**Table 2:** Evidence status of the key links in the proposed model. Tiers: 1, human ASD data; 2, other human data; 3, animal, organoid, or in vitro data; 4, hypothesis. Tier labels refer to the link as named in the first column; rows ending in ASD risk or neurodevelopment provide no evidence on regression, symptom severity, or diagnostic timing.

Link in the Proposed Model	Tier (s)	Best Available Evidence	Main Limitations	Status for the Model (Section)
Oxidative-stress alterations in ASD	1	Reduced glutathione and altered oxidative-damage markers reported in ASD samples (review-level sources) [6,7]	Cross-sectional; cannot show cause; diet, medication, and illness confound	Motivates but does not test mediation (Section 3)
Air pollution → ASD risk	1	Meta-analyses and large cohorts or consortia [58,59,60,61]; windows in late gestation and the second postnatal year	Modest effects; socioeconomic confounding and ascertainment; leading components differ between cohorts	Risk only; regression untested (Section 3.2.3)
Micro- and nanoplastics → neurodevelopment	2–3	Detection in human placenta and brain [33,34,35,36]; animal and organoid effects [39,40,41,44,46]; one occupational severity study in ASD [45]	No human ASD-outcome study; method variability; uncertain dose relevance	Plausible route; Tier 4 for ASD (Section 3.2.1)
EDCs (BPA, phthalates, OPEs) → ASD	1 (mixed), 3	BPA–aromatase pathway in one cohort plus mice [50]; OPE signal in ECHO [51]; null or mixed phthalate cohorts [53,54,55]	Inconsistent across cohorts; mixtures; sex-specific effects; general-behavior outcomes	Mixed (Section 3.2.2)
Tire-wear 6PPD-quinone → fetal brain	3	Placental transfer and fetal-brain accumulation in pregnant mice [57]	No human exposure-to-ASD data	Hypothesis (Section 3.2.2)
Metals, pesticides, persistent pollutants → ASD	1	Case–control and cohort studies [65,66]; household insecticides [67]; third-trimester signal in boys from dentine biomarkers [69]	Low pooled certainty [66]; retrospective designs; single-cohort biomarker finding	Risk only (Section 3.2.4)
Ultraprocessed food → oxidative burden	2, 4	One inpatient trial (energy intake) [70]; cross-sectional oxidative and inflammatory biomarkers [71,72,73]	No pregnancy or ASD data; partial independence from adiposity	Extrapolation (Section 3.2.5)
Psychosocial stress and ACEs → oxidative or inflammatory burden	1, 2–3	Higher ACE burden in autistic youth [103]; catatonia linked to stressful events [17]; epigenetic and immune findings in non-ASD cohorts [29,31]	No study of stress predicting regression; reverse causation	Hypothesis (Section 3.1 and Section 6)
Postnatal infection → abrupt change or regression	1–2	PANS/PANDAS series and incidence estimates [77,79]; influenza–neuropsychiatric hospitalization study (general pediatric) [82]	Diagnostic uncertainty; prevalence in ASD unknown; alternative explanations for timing	Plausible; ASD regression untested (Section 3.3)
Maternal immune activation or autoimmune disease → ASD	1, 3	Population studies (about 30% higher odds with maternal autoimmune disease) [8]; animal, organoid, and cerebroid models [8,91]	Familial confounding [84]; mechanisms mostly experimental	Prenatal risk (Section 4)
FRAA and cerebral folate deficiency in ASD	1	Replicated FRAA prevalence in ASD [8,92]; small randomized folinic acid trials [8,93]	Small, short trials; homocysteine–oxidative link and link to regression untested	Biomarker established; mediation Tier 4 (Section 4.3)
Microbiome → B9/B12 → one-carbon and antioxidant capacity	3; 1 (null)	Genome surveys of folate synthesis [94]; animal data; prospective infant cohort without early differences [97]; small probiotic trial [99]	Confounding and reverse causation in case–control data; compositional change without behavioral change	Chain as a whole Tier 4 (Section 5)
Mitochondrial vulnerability preceding regression	1 (single study)	One study of the order of mitochondrial and immune changes by regression status [7]	Not replicated; retrospective regression classification	Provisional (Section 3.1)
Endocannabinoid signaling	1 (small), 3	Lower circulating anandamide and related lipids in ASD children [87,89]; rodent-model modulation [85]	Small studies; model-dependent; clinical cannabinoid data uncontrolled	Candidate modulator (Section 3.5)
Cumulative-burden threshold model	4	None direct	No study measures burden, buffering, and timing together	Hypothesis; predictions in Section 8.5
Sex-linked oxidative buffering	3, 4	Estrogen antioxidant effects in experimental systems; sex differences in HPA reactivity; sex-biased phenotypes in Ube3a-overexpressing mice (plausibility only) [112,113]	No ASD timing data; indistinguishable from masking with current data	Hypothesis (Section 7.3)
Masking and diagnostic criteria → sex-disparate timing	1	Registry and records data [109,110,111]; clinical literature [114,115]	Camouflaging measured indirectly	Best-supported explanation (Section 7.2)

## 9. Limitations and Required Future Evidence

This paper is a theoretical synthesis, not a report of new empirical data, and its central contribution is the proposed mechanistic connection between studies rather than a demonstrated causal pathway. A direct test of the model would require a prospective cohort design measuring oxidative stress markers, gut microbiome composition, B9/B12 status, cortisol, and detailed environmental exposure history simultaneously in children followed from early childhood through adolescence, with sufficient statistical power to compare those who experience early regression, late regression, late diagnosis, and typical early diagnosis. No such study currently exists.

Alternative explanations that do not require this model must also be acknowledged. As noted in Section 2.5 and Section 7.2, late diagnosis may be substantially explained by evolving criteria, clinical awareness, and masking alone. The “gained diagnosis” trajectory identified by Elias and Lord [18] could reflect measurement or instrument sensitivity changes across development rather than a true biological process. These alternatives are not mutually exclusive with the model proposed here, but a rigorous test of this paper’s hypothesis would need to demonstrate an oxidative/exposure signal above and beyond what these more established explanations already account for.

The review method itself has limits. A narrative search by one author is vulnerable to selection bias and to favoring evidence that fits the framework, and we did not apply a formal risk-of-bias tool (Section 1.2). The nonspecificity of oxidative-stress markers, the uncertain direction of causation, and the retrospective identification of regression are addressed by the design requirements in Section 8.5. The model also does not address the large genetic contribution to ASD, including syndromic forms in which regression is part of the clinical picture, and many cases of regression or late diagnosis may have no identifiable environmental contribution.

We have tried to place this paper in relation to the broader literature, including findings that argue against or limit the model: the null prospective microbiome result, the possibility that maternal immune activation associations reflect familial confounding, the low certainty of pooled pollutant evidence, and the strength of the diagnostic and masking explanations for late diagnosis and for the sex difference in timing. Our earlier reviews addressed ASD risk generally; the contribution here is the focus on timing and trajectory, with operational definitions and predictions (Section 8.5) that can be tested and, if the predictions fail, refuted.

## 10. Conclusions

We have asked whether a cumulative oxidative and inflammatory burden, shaped by environmental exposure, infection and autoimmune activity, and psychosocial stress, mediated in part by the gut microbiome and one-carbon metabolism, and modified by genetic susceptibility and individual buffering capacity, could contribute to some autism trajectories (early and late regression and gradual increases in severity) and, more tentatively, to sex differences in diagnostic timing. Based on the current evidence the idea is plausible and testable but unproven. Each exposure domain is associated with ASD risk or acts on relevant mechanisms in experimental systems, but none has been linked to regression or late diagnosis, oxidative stress may be a consequence or correlate rather than a cause, and diagnostic and recognition processes account for much of the late-diagnosis and sex-timing data. We have therefore presented the model as a hypothesis, defined its terms, derived predictions that could refute it (Section 8.5), and graded the evidence for each link (Table 2). Reducing avoidable environmental exposures and supporting families are reasonable on independent grounds and do not depend on this model, but the present evidence does not support specific clinical or preventive interventions for regression or late diagnosis, and nothing here implies that parents or families cause autism. Progress depends on the prospective, sex-stratified cohorts described in Section 9.

## Data Availability

No new data were created or analyzed in this study. Data sharing is not applicable to this article.

## Abbreviations

The following abbreviations are used in this manuscript:

2-AG	2-Arachidonoylglycerol
ACE	Adverse Childhood Experience
ADDM	Autism and Developmental Disabilities Monitoring (Network)
ADHD	Attention-Deficit/Hyperactivity Disorder
AEA	Anandamide
ASD	Autism Spectrum Disorder
AUC	Area Under the Curve
BPA	Bisphenol A
CASSQ	Childhood Adversity and Social Stress Questionnaire
CB1	Cannabinoid Receptor Type 1
CB2	Cannabinoid Receptor Type 2
CDC	Centers for Disease Control and Prevention
CFD	Cerebral Folate Deficiency
CHARGE	Childhood Autism Risks from Genetics and the Environment (cohort study)
CNS	Central Nervous System
CNV	Copy Number Variant
CRP	C-Reactive Protein
DSM-5	Diagnostic and Statistical Manual of Mental Disorders, Fifth Edition
eCB	Endocannabinoid
ECHO	Environmental influences on Child Health Outcomes (consortium)
EDC	Endocrine-Disrupting Chemical
FAAH	Fatty Acid Amide Hydrolase
FRα	Folate Receptor Alpha
FRAA	Folate Receptor Alpha Autoantibody
GABA	Gamma-Aminobutyric Acid
HPA	Hypothalamic–Pituitary–Adrenal (axis)
hs-CRP	High-Sensitivity C-Reactive Protein
IL-6	Interleukin-6
IL-17A	Interleukin-17A
IQ	Intelligence Quotient
LPS	Lipopolysaccharide
MAGL	Monoacylglycerol Lipase
MAR	Maternal Autoantibody-Related (autism)
MARBLES	Markers of Autism Risk in Babies—Learning Early Signs (cohort study)
MIA	Maternal Immune Activation
MMR	Measles, Mumps, and Rubella (vaccine)
MNP	Micro- and Nanoplastic
MTHFR	Methylenetetrahydrofolate Reductase
NIH	National Institutes of Health
OCD	Obsessive–Compulsive Disorder
OPE	Organophosphate Ester
PANDAS	Pediatric Autoimmune Neuropsychiatric Disorders Associated with Streptococcal infections
PANS	Pediatric Acute-onset Neuropsychiatric Syndrome
PCB	Polychlorinated Biphenyl
PM2.5	Fine Particulate Matter (≤2.5 µm in Diameter)
PRISMA	Preferred Reporting Items for Systematic Reviews and Meta-Analyses
RFC	Reduced Folate Carrier
SCFA	Short-Chain Fatty Acid
SPARK	Simons Foundation Powering Autism Research for Knowledge (cohort study)
TLR4	Toll-Like Receptor 4
TNF-α	Tumor Necrosis Factor-alpha
UPF	Ultraprocessed Food
